# Shoc2 recognizes bacterial flagellin and mediates antibacterial Erk/Stat signaling in an invertebrate

**DOI:** 10.1371/journal.ppat.1010253

**Published:** 2022-01-24

**Authors:** Bao-Rui Zhao, Xin-Xin Wang, Xian-Wei Wang

**Affiliations:** 1 Shandong Provincial Key Laboratory of Animal Cells and Developmental Biology, School of Life Sciences, Shandong University, Qingdao, China; 2 State Key Laboratory of Microbial Technology, Shandong University, Qingdao, China; 3 Laboratory for Marine Biology and Biotechnology, Qingdao National Laboratory for Marine Science and Technology, Qingdao, China; Uppsala University, SWEDEN

## Abstract

Flagellin is a key bacterial virulence factor that can stimulate molecular immune signaling in both animals and plants. The detailed mechanisms of recognizing flagellin and mounting an efficient immune response have been uncovered in vertebrates; however, whether invertebrates can discriminate flagellin remains largely unknown. In the present study, the homolog of human SHOC2 leucine rich repeat scaffold protein in kuruma shrimp (*Marsupenaeus japonicus*), designated MjShoc2, was found to interact with *Vibrio anguillarum* flagellin A (FlaA) using yeast two-hybrid and pull-down assays. MjShoc2 plays a role in antibacterial response by mediating the FlaA-induced expression of certain antibacterial effectors, including lectin and antimicrobial peptide. FlaA challenge, via MjShoc2, led to phosphorylation of extracellular regulated kinase (Erk), and the subsequent activation of signal transducer and activator of transcription (Stat), ultimately inducing the expression of effectors. Therefore, by establishing the FlaA/MjShoc2/Erk/Stat signaling axis, this study revealed a new antibacterial strategy in shrimp, and provides insights into the flagellin sensing mechanism in invertebrates.

## Author summary

Flagellin sensing has been proven as a general antibacterial strategy. Recognition of bacterial flagellin by the transmembrane receptor toll like receptor 5 (TLR5) leads to the activation of nuclear factor kappa B (NF-κB) pathway and induction of proinflammatory cytokines, while recognition by the intracellular nucleotide-binding leucine-rich (NLR) receptor leads to caspase-activation and cytokines-expression. Although flagellin is an effective immune stimulator that induces antimicrobial peptides in *Drosophila* and in crustaceans, how an invertebrate host senses flagellin and mounts an immune response is poorly understood. Here, we used the flagellin (FlaA) from *Vibrio anguillarum*, a pathogen of shrimp, as a bait protein to screen a yeast two-hybrid library derived from kuruma shrimp (*Marsupenaeus japonicus*). We found a scaffold protein, MjShoc2, able to interact with FlaA. We also found that FlaA could effectively induce the expression of certain recognized antibacterial effectors in shrimp depending on MjShoc2. We revealed that extracellular regulated kinase (Erk) phosphorylation occurred downstream of FlaA/MjShoc2, and led to signal transducer and activator of transcription (Stat) activation, resulting in transcription of certain effectors. Therefore our study provides new insights into the FlaA-induced molecular immunity in invertebrates.

## Introduction

Many bacterial species have a flagellum [1]. Flagellum contributes to bacterial pathogenesis by participating in multiple stages throughout the infection process, including adherence and colonization, biofilm development, secretion of other virulence effectors, further penetration through tissues, and modulation of host immune responses [2,3]. Flagellin is the most abundant component of a flagellum [4]. The significance of flagellins in bacterial pathogenesis have been fully explored. For example, *Vibrio anguillarum* genome encodes five flagellin genes. Mutation of flagellin A (FlaA) of the NB10 strain led to a 50% decrease in motility and a 700-fold decrease in the bacteria’s infection ability, while deletion of FlaD or FlaE did not alter motility, but suppressed the pathogenicity markedly [5,6]. These data suggested the multifactorial involvement of flagellins in *V*. *anguillarum* virulence. In addition, flagellins possess good immunogenicity. Low concentrations of flagellin can stimulate pro-inflammatory signaling [7,8]. Indeed, flagellins have been used as common vaccine adjuvants to induce both cellular and humoral immune responses [9–11]. Therefore, sensing flagellin and initiating immune responses could be an effective strategy to resist infection.

The pattern recognition receptors (PRR) recognizing flagellin have been identified in mammals. The major receptor for extracellular flagellin is toll like receptor 5 (TLR5) [12]. Recognition of *Salmonella* flagellin (FliC) by mammalian TLR5 leads to MyD88-mediated signaling, activation of NF-κB and MAPK pathways, and production of proinflammatory cytokines, including TNF-α, IL-1, and IL-8, in monocytes, epithelial cells, and fibroblasts [13–15]. When flagellin is present in the cytosol, it is detected by intracellular receptors such as NAIP5/6 [16]. Recognition of flagellin from *Legionella pneumophila* by NAIP5/6 leads to the recruitment of NLRC4, and the formation of the Flagellin-NAIP5/6-NLRC4 inflammasome. The inflammasome recruits and activates caspase 1 directly, and induces the expression of IL-1β and IL-18 [17,18].

Studies show that flagellin could also induce certain molecular immune responses in invertebrates. For example, cecropin expression could be significantly induced in *Drosophila* mbn-2 cells after treatment with *Bacillus thuringiensis* flagellins [8]. Fusion of *S*. *typhimurium* Flagellin2 with a white spot syndrome virus vaccine enhanced the protective effect and induced NF-κB activation in freshwater prawn *Palaemon paucidens* [19]. These findings support that invertebrates possess the ability to sense bacterial flagellins and initiate downstream molecular immunity. However, how invertebrate host recognizes bacterial flagellin remains unknown.

In this study, we performed a yeast two-hybrid screening to identify the potential flagellin-recognizing proteins from kuruma shrimp (*Marsupenaeus japonicus*), which is an important aquaculture species that has long suffered from infections by flagellated *Vibrio* spp. [20,21]. By revealing the function of the flagellin-recognizing protein in antibacterial response, and establishing the signaling axis induced by flagellin, we aimed to uncover how shrimp recognizes bacterial flagellin and mount an efficient immune response. The identification and characterization of the flagellin-recognizing protein in shrimp would shed light on flagellin-sensing mechanism in invertebrates.

## Results

### MjShoc2 recognizes and responds to intracellular V. anguillarum FlaA

**Fig 1:**
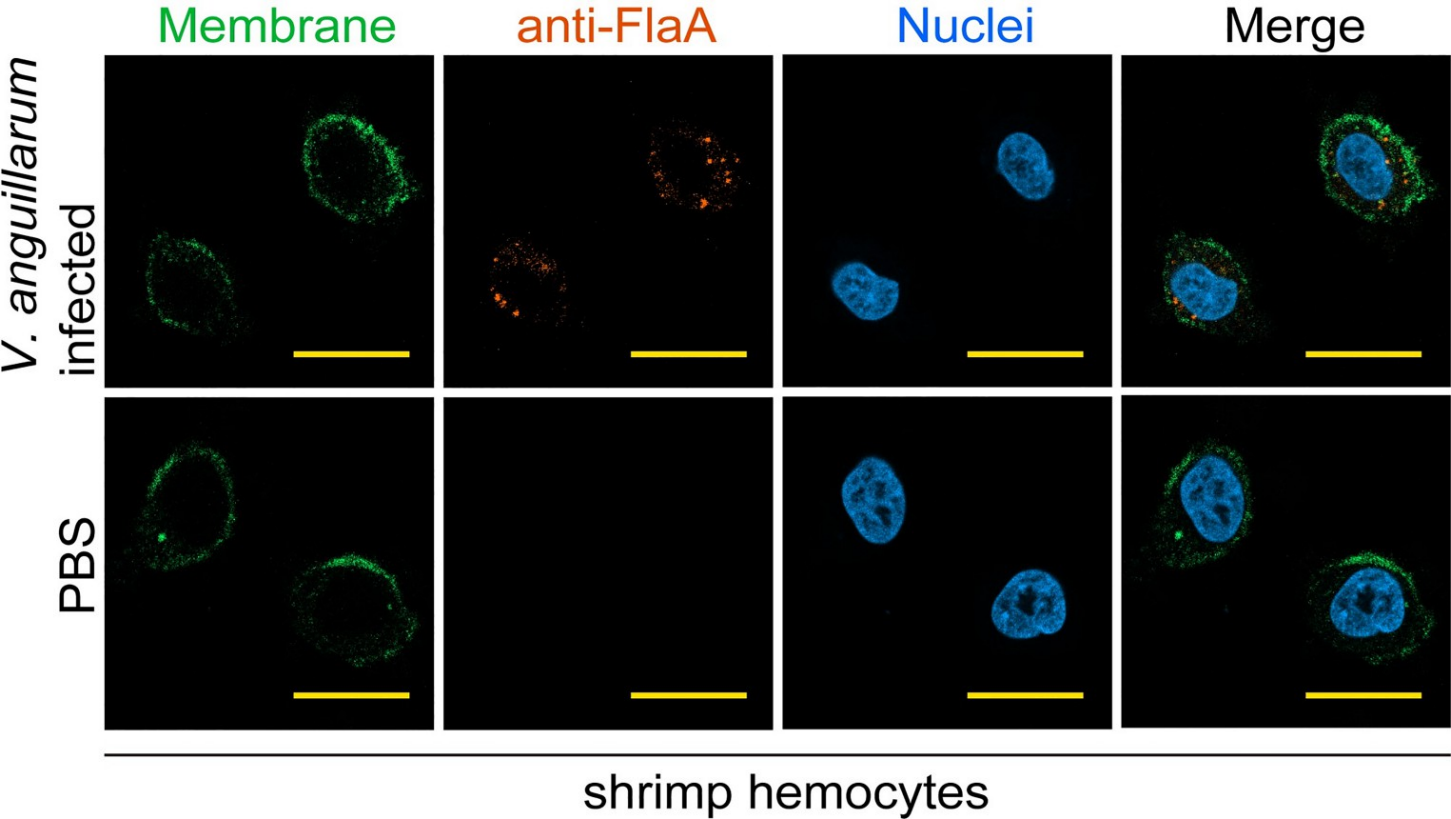
Presence of FlaA in the hemocytes from. *V*. *anguillarum*-infected shrimp. Hemocytes were collected at 6 h after *V*. *anguillarum* infection. FlaA was detected using rabbit anti-FlaA antibodies and goat anti-rabbit Alexa Fluor 594, and cell membrane was visualized with a Dio membrane probe. DAPI was used to stain the nuclei. Scale bar = 10 μm. The data are representative of two independent repeats.

**Fig 2:**
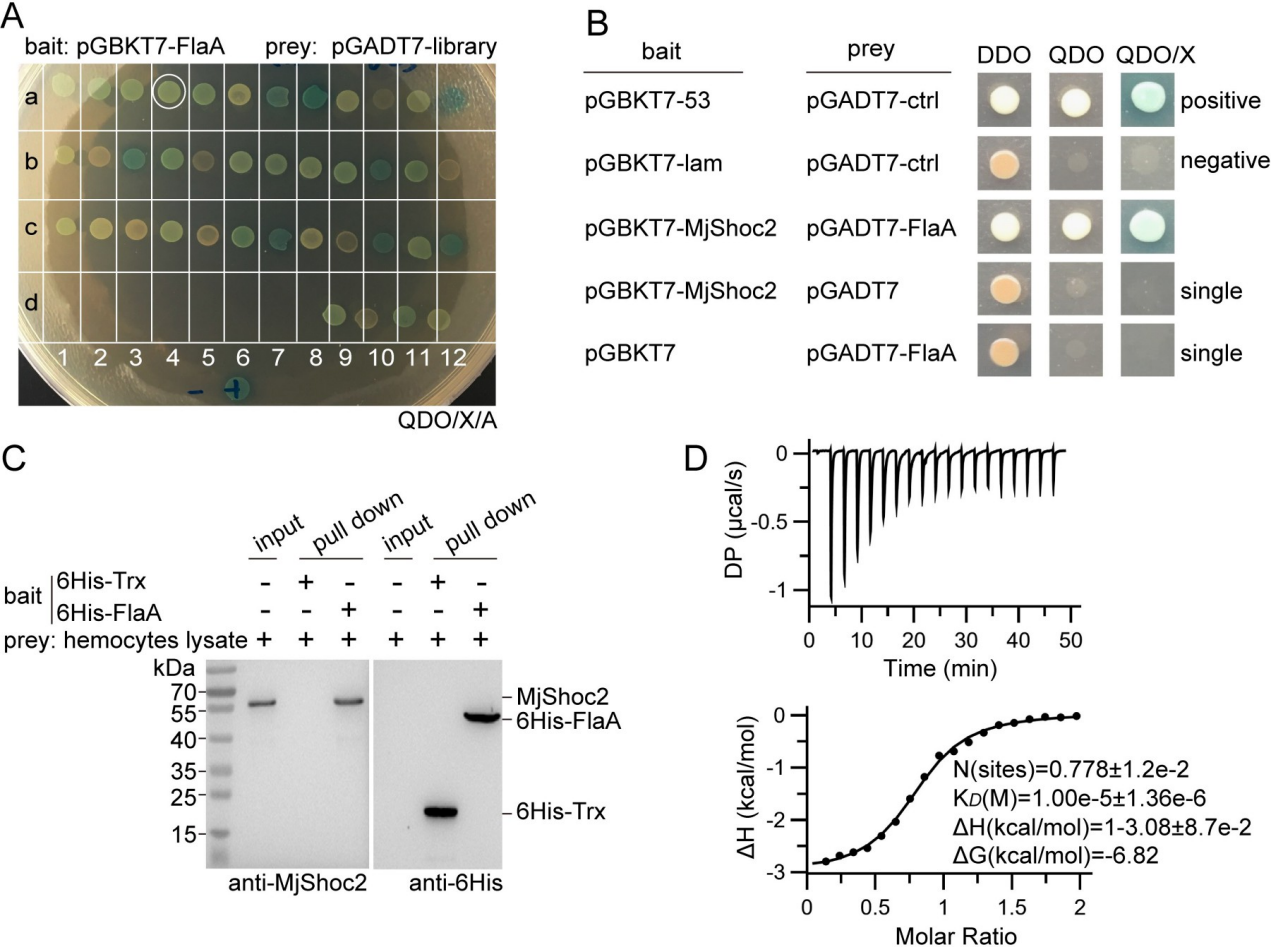
Identification and verification of MjShoc2-FlaA interaction. (A) Y2H identification of FlaA-interacting proteins. FlaA was used as the bait to screen a Gateway AD library derived from hemocytes, hepatopancreas, gills and intestine from kuruma shrimp *M*. *japonicus*. (B) Pairwise verification of the MjShoc2-FlaA interaction. Full length cDNA of *MjShoc2* and *FlaA* was cloned into the pGBKT7 vector and pGADT7 vector, respectively. The paired plasmids, as indicated, were co-transformed into yeast cells. Data are representative of two repeats. (C) Determination of interaction between FlaA and native MjShoc2. His-tagged FlaA or control tag was used to pull native MjShoc2 from shrimp hemocytes homogenate. Eluate from Ni-NTA resin was analyzed using western blotting with anti-MjShoc2 antibodies and anti-6His antibodies. Data are representative of two repeats. (D) Characterization of interaction between FlaA and rMjShoc2 by ITC assay. rFlaA in the syringe was injected into cell containing rMjShoc2. Upper, raw plot; lower panel, integrated heat plot. Data are representative of three independent repeats.

Immunocytochemical analysis using FlaA antibody showed positive signal in the hemocytes from *V*. *anguillarum*-infected shrimp (Fig 1), suggesting the presence of FlaA or its fragment in host cell cytoplasm after infection. Therefore, *V*. *anguillarum* FlaA was used as the bait to screen a Gateway AD yeast two-hybrid library derived from kuruma shrimp to identify the proteins that bind flagellin. As shown in Fig 2A, the screening yielded a total of 40 prey proteins; however, nine of them (a10, b2, b5, b12, c3, c5, c8, c9, and d10) were regarded as negative because of weak growth on QDO plates. The remaining clones were sequenced and the results obtained were listed in S1 Table. Among the candidates, one clone (a4), which encoded the homolog of the SHOC2 LRR scaffold protein, designated MjShoc2, attracted our attention because it comprised of long stretch of LRRs, which have been proven as key modules to interact with flagellins. Pairwise analysis was then performed to check the interaction between full length MjShoc2 and FlaA, and the results again demonstrated a specific interaction (Fig 2B).

To verify these results, a pull-down assay was performed. The result showed FlaA could interact with native MjShoc2 from the hemocytes lysate (Fig 2C), thus confirming the MjShoc2-FlaA interaction. In addition, an isothermal titration calorimetry (ITC) assay was performed to characterize the interaction between FlaA and recombinant MjShoc2. As shown in Fig 2D, the interaction mainly fit the one set of sites model, and the K_*D*_ was about 10 μM.

**Fig 3:**
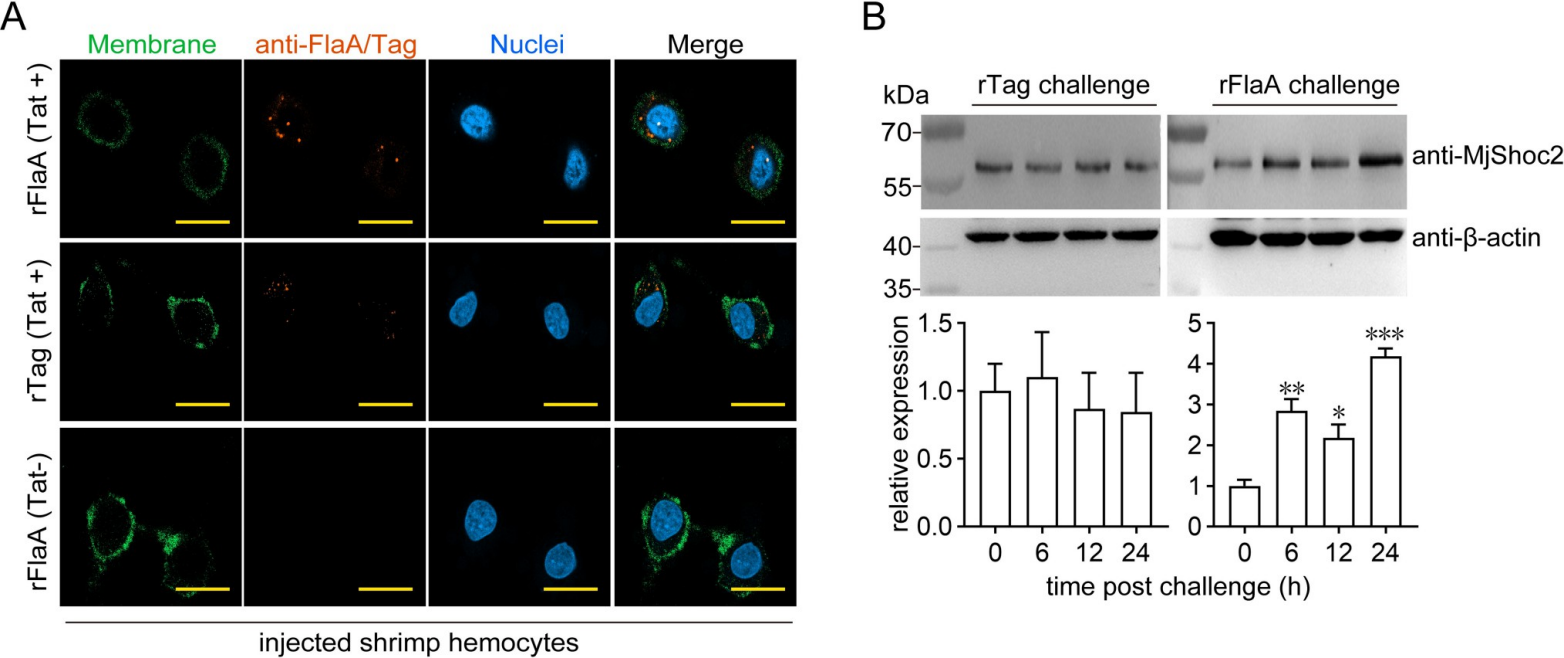
Expression profiles of MjShoc2 after FlaA challenge. (A) Entering into hemocytes cytosol of rFlaA fused with Tat cell-penetrating peptide. Hemocytes were collected at 6 h after rFlaA injection. Rabbit anti-FlaA antibodies and goat anti-rabbit Alexa Fluor 594 was used to visualize signal and Dio probe was used to stain the cell membrane. DAPI was used to stain the nuclei. Scale bar = 10 μm. The data are representative of two independent repeats. (B) MjShoc2 protein expression after injection of 10 μg of rFlaA or control tag in hemocytes. All samples were originated from at least five shrimp. Upper panel, representative western blotting result; lower panel, quantification and statistical analysis of the blotting data from three repeats. *, 0.01 < *p* < 0.05; **, 0.001 < *p* < 0.01; ***, *p* < 0.001.

Above data proved that MjShoc2 was a new *V*. *anguillarum* flagellin-recognizing protein; therefore, the expression profiles of MjShoc2 were next studied to check whether it was involved in FlaA-induced immunity. Recombinant FlaA (rFlaA), which was fused with the cell-penetrating peptide Tat 49–57 [22] and able to penetrate through cell membrane and enter the cytoplasm (Fig 3A), was used to imitate the monomeric flagellin present in the cytosol to challenge the shrimp. As shown in Fig 3B, MjShoc2 expression was induced by rFlaA challenge. The increased MjShoc2 was distributed in the cytoplasm (S1 Fig). The induced expression suggested the correlation of MjShoc2 to host-*V*. *anguillarum* interaction.

### MjShoc2 functions in antibacterial response by mediating the expression of FlaA-induced antibacterial effectors

**Fig 4:**
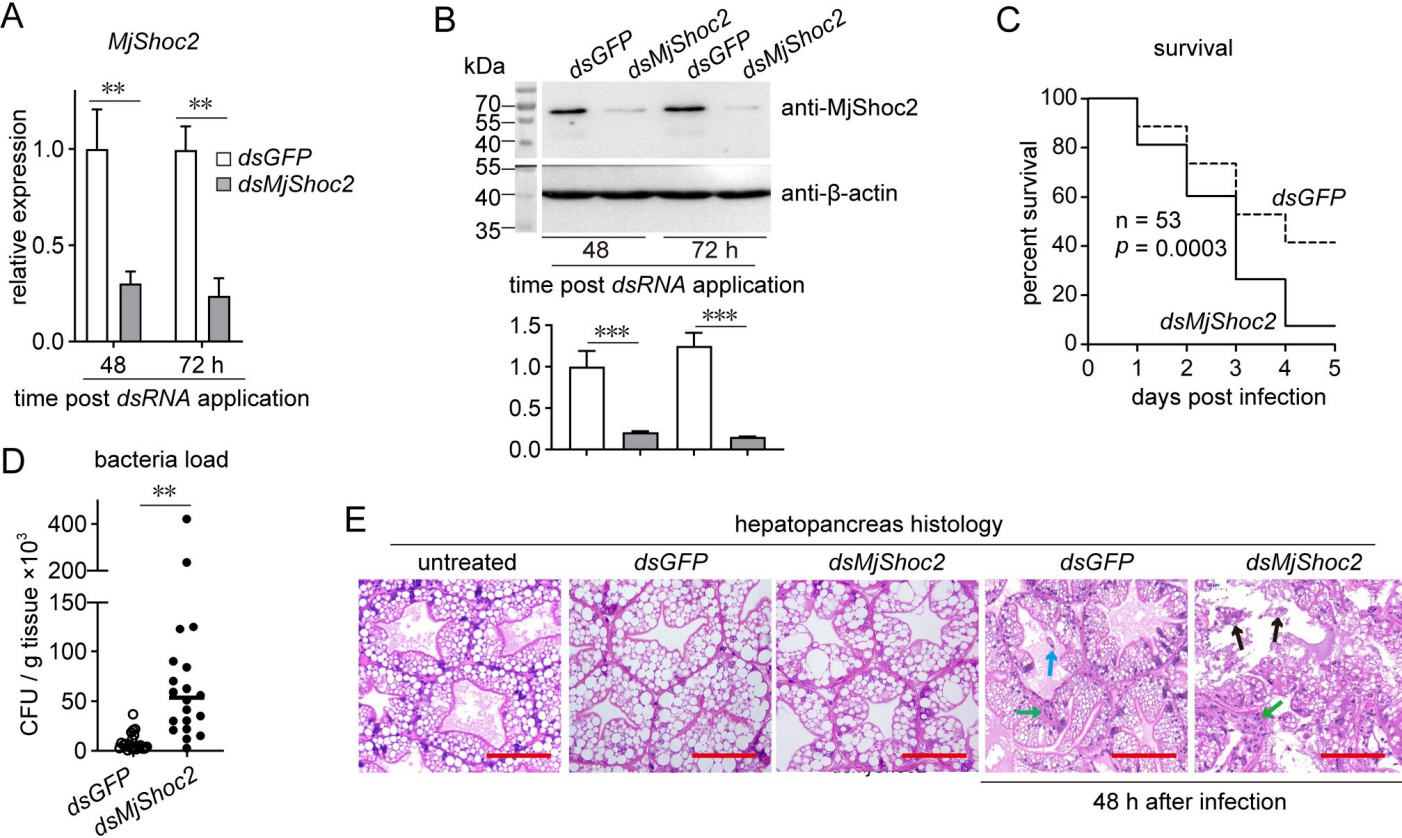
Role of MjShoc2 against *V*. *anguillarum* infection. (A) *MjShoc2* expression after RNAi. dsRNA was injected into shrimp hemocoels at a dose of 5 μg per gram of body weight. RNAi efficiency was detected at 48 h and 72 h after dsRNA application. Data are mean ± SD from three repeats. Each sample originated from at least five animals. (B) MjShoc2 protein expression after RNAi. Upper panel, representative western blotting result; lower panel, quantification and statistical analysis of the blotting data from three repeats. Each sample originated from at least five animals. (C) Protective role of MjShoc2 against *V*. *anguillarum*. *V*. *anguillarum* infection was performed at 24 h after dsRNA application, and the survival rate was recorded for 5 d. The data are representative of two independent repeats. (D) Effect of MjShoc2 knockdown on hepatopancreas bacterial load after infection. Hepatopancreas homogenate was plated onto agar plates containing the *V*.*anguillarum*-resistant antibiotics to determine bacterial counts. The data are representative of two independent repeats. (E) Morphological analysis of heapatopancreas showing the protective role of MjShoc2. Tissues were sectioned and stained with H&E. Arrows show the sloughing of hepatopancreatic cells (blue arrow), hemocytes infiltration (green arrow), and tissue damage (black arrow). Scale bar = 50 μm. The data are representative of two independent repeats. **, 0.001 < *p* < 0.01; ***, *p* < 0.001.

To determine the role of MjShoc2 in antibacterial response, RNAi was performed to knockdown *MjShoc2* expression to determine the effect on host immunity. *dsMjShoc2* treatment suppressed *MjShoc2* expression (Fig 4A) and decreased the MjShoc2 protein amount (Fig 4B) until the third day after dsRNA application, but did not led to shrimp death (S2 Fig). *V*. *anguillarum* was then used to infect the treated shrimp, and the results showed that the survival rate of the *MjShoc2* knockdown group was significantly lower than that of the control group. About 50% of the animals were alive at 5 d after *V*. *anguillarum* infection in the control group, while the survival rate in the *MjShoc2* knockdown group was less than 10% (Fig 4C). In addition, the counts of externally injected bacteria were significantly higher in *MjShoc2* knockdown shrimp than in the control shrimp (Fig 4D).

The shrimp hepatopancreas was collected for histological analysis. As shown in Fig 4E, *MjShoc2* knockdown did not lead to obvious change in tissue morphology. However, after bacterial infection, sloughing of hepatopancreatic cells and hemocytes infiltration, which are the typical histopathological alterations of a hepatopancreas infected with bacterial pathogens, were observed in the control group, while the tissue damage was more serious in the *MjShoc2* knockdown group. Taken together, the above data suggested that MjShoc2 was involved in the antibacterial immunity.

**Fig 5:**
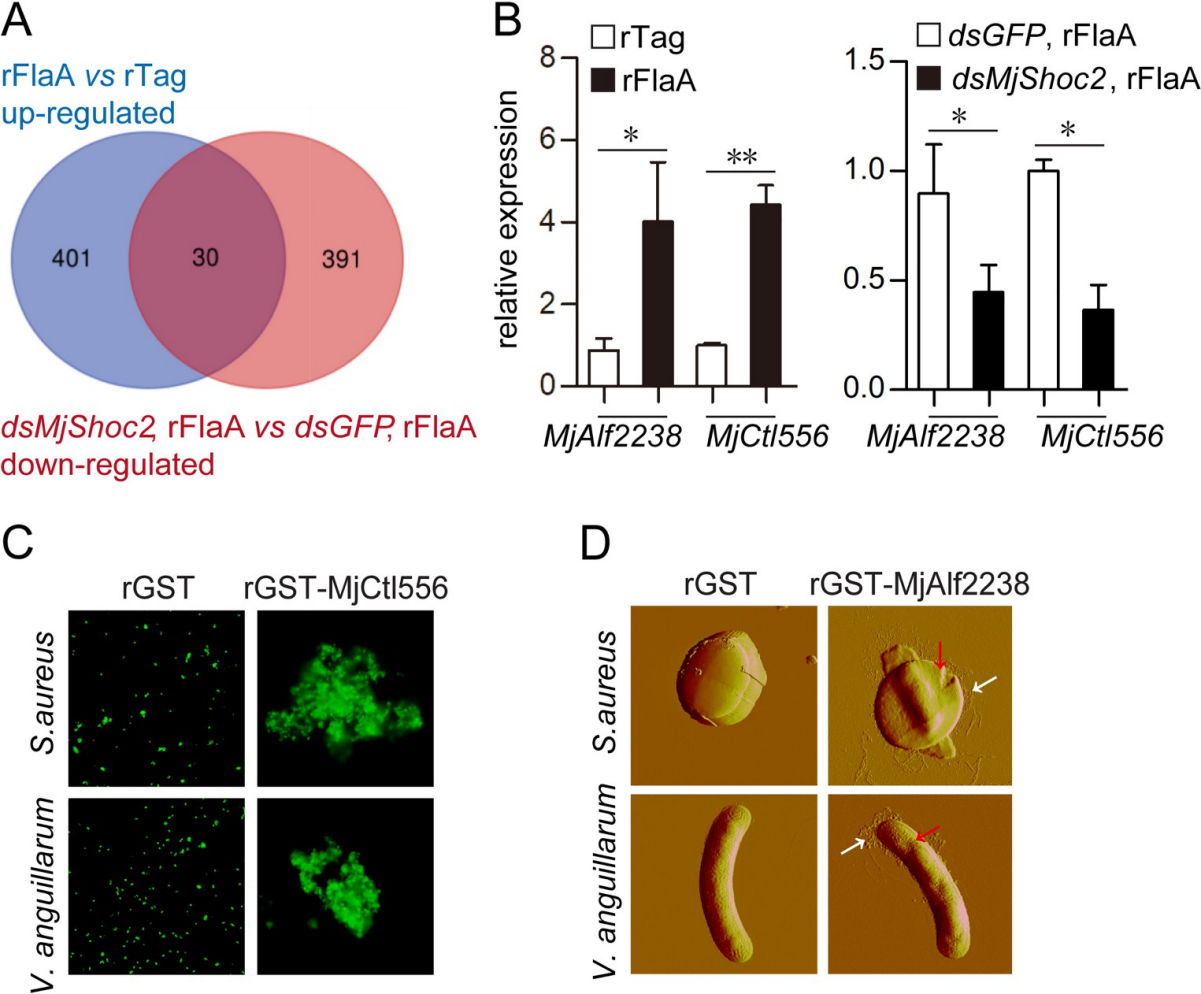
Identification of FlaA/MjShoc2-induced genes. (A) Venn diagram of the overlap of the differentially expressed genes. Untreated shrimp or dsRNA pre-treated shrimp were injected with 5 μg of rTag or rFlaA. Total RNAs from hemocytes and intestine were collected 6 h later. Three biological replicates were performed. Equal amounts of the RNAs from hemocytes and intestine of each replicate were mixed for the transcriptome sequencing. Only the genes with an FPKM ≥ 2 and a fold change ≥ 2 were considered as valid. Each group consisted of at least 30 animals. (B) Validation of the transcriptome sequencing result by qRT-PCR in hemocytes. Data are shown as the mean ± SD from three independent repeats. Each sample originated from at least five animals. *, 0.01 < *p* < 0.05; **, 0.001 < *p* < 0.01. (C) Bacterial agglutination caused by MjCtl556. The bacteria were labeled with FITC, and observed under a fluorescence microscope. Data are representative of three repeats. (D) Antimicrobial activity of MjAlf2238. Bacteria was treated with 25 μM protein and atomic force microscopy images were taken. Red arrow, disruption of bacterial integrity; white arrow, leakage of intracellular materials. Data are representative of two repeats.

Next a *de novo* transcriptomic analysis was performed to identify the genes regulated by MjShoc2 after FlaA challenge. The sequencing aimed to obtain the targets whose expression was induced by FlaA challenge and, at the same time, whose induction was suppressed after *MjShoc2* knockdown. Venn analysis of differentially expressed genes in the two parts generated 30 candidates (Fig 5A), including a set of immunity-related genes (S2 Table). Special attention was paid onto two members from the C-type lectin (Ctl) and anti-lipopolysaccharides factor (Alf) families, which were previously proven to be involved in the antibacterial responses by acting as agglutinins and antimicrobial peptides [23,24]. The high throughput sequencing results were then verified using qRT-PCR (Fig 5B). The consistent results between sequencing and qRT-PCR suggested that MjShoc2 indeed regulates the FlaA-induced expression of MjCtl556 and MjAlf2238. Next the specific roles of MjCtl556 and MjAlf2238 in antibacterial immunity were investigated. MjCtl556 could agglutinate bacteria (Fig 5C), while MjAlf2238 exhibited direct antimicrobial activity by destroying the bacterial integrity (Fig 5D).

### MjShoc2 mediates FlaA-induced expression of antibacterial effectors in a Stat-dependent manner

**Fig 6:**
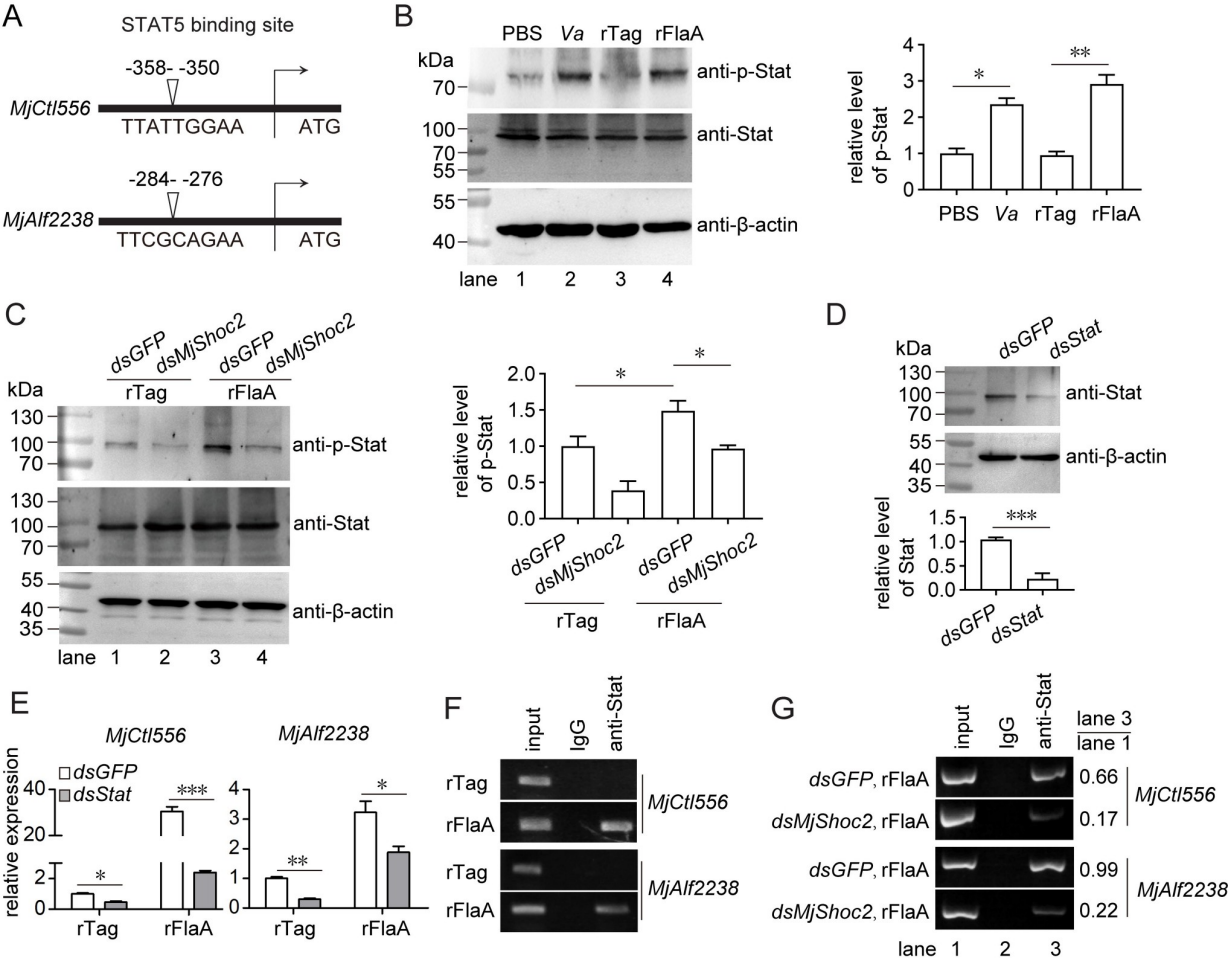
Regulation of FlaA-induced antibacterial effectors by Stat. (A) Analysis of the promoters of *MjCtl556* and *MjAlf2238*. The 5′ untranslated regions were obtained and analyzed using the online Promoter 3.0 tool and JAPAR tool. (B) Stat phosphorylation induced by *V*. *anguillarum* or rFlaA, with PBS or rTag as control, respectively. Stat phosphorylation was detected using a human Phospho-STAT5A-Y694 antibody. Left panel, representative blotting result; right panel, quantification and statistical analysis of the blotting data from three repeats. (C) Influence of MjShoc2 knockdown on Stat-activation caused by FlaA. rFlaA injection was performed 24 h after dsRNA application, and Stat phosphorylation was detected another 6 h later. Left panel, representative blotting result; right panel, quantification and statistical analysis of the blotting data from three repeats. (D) RNAi efficiency of Stat. Expression was detected by Western blotting at 24 h after dsRNA application. Upper panel, representative blotting result; lower panel, quantification and statistical analysis of the blotting data from three repeats. (E) Influence of *Stat* knockdown on the induction of *MjCtl556* and *MjAlf2238* expression by FlaA. rFlaA injection was performed 24 h after dsRNA application, and the expression levels were detected 6 h later. Data are mean ± SD from three repeats. (F) ChIP assay showing the binding of Stat to *MjCtl556* and *MjAlf2238* promoters. Shrimp were injected with rFlaA or the tag. The hemocytes were collected as the pool for the ChIP assay at 6 h later. The immunoprecipitates were analyzed using RT-PCR with primers specific for the fragments containing the STAT5 binding sites. Data are representative of three repeats. (G) Influence of *MjShoc2* knockdown on FlaA-induced binding of Stat to *MjCtl556* and *MjAlf2238* promoters. The number shows relative ratio of the band intensity (anti-Stat/input) of each panel. Data are representative of three repeats. Each sample originated from at least five animals. *, 0.01 < *p* < 0.05, **, 0.001< *p* < 0.01, ***, *p* < 0.001.

To reveal how FlaA-MjShoc2 regulates the transcription of *MjCtl556* and *MjAlf2238*, the promoter sequences of two genes were cloned and the possible transcription factor binding sites were predicted. As shown in Figs 6A and S3, a consensus STAT5 responsive element (TTCT/CNA/GGAA) was identified in the upstream sequences of both *MjCtl556* and *MjAlf2238*, suggesting the possible transcription regulation of these two genes by shrimp Stat, whose DNA-binding domain showed 60% identity to that of human STAT5.

Afterwards, Stat phosphorylation level was determined after *V*. *anguillarum* infection or FlaA challenge, and the results showed that Stat phosphorylation was induced after both treatments, suggesting that Stat-activation might play a role in the antibacterial response (Fig 6B). To check whether MjShoc2 was related to FlaA-induced Stat-activation, the challenge was performed in the *MjShoc2* pre-silenced shrimp. As shown in Fig 6C, the increase of Stat phosphorylation induced by FlaA (lane 3 *vs* lane 1) was suppressed when *MjShoc2* expression was silenced (lane 4 *vs* lane 3), suggesting MjShoc2 was essential for FlaA-induced Stat-activation.

The above data suggested the probable existence of a FlaA/MjShoc2/Stat axis; therefore, whether the FlaA/MjShoc2-induced expression of antibacterial effectors was dependent on Stat was investigated. Stat expression was silenced using RNAi (Fig 6D), which resulted in the suppression of FlaA-induced expression of *MjCtl556* and *MjAlf2238* (Fig 6E). Moreover, a chromatin immunoprecipitation (ChIP) assay was performed to determine whether Stat could bind the fragments containing the STAT5 responsive element. As shown in Fig 6F, for both *MjCtl556* and *MjAlf2238*, positive signals were detected from the immunoprecipitates only when using the FlaA-challenged hemocytes as the pool for ChIP. This suggested that Stat transcriptionally regulate expression of antibacterial effectors directly in the presence of FlaA challenge. To further reveal the contribution of MjShoc2 in the transcription of effectors, ChIP assay was next performed after dsRNA application and FlaA challenge. As shown in Fig 6G, MjShoc2 knockdown led to decreased amount of *MjCtl556* and *MjAlf2238* fragments in the immunoprecipitates of Stat antibodies, further suggesting that MjShoc2 regulated Stat-dependent transcription of two effectors. Thus, these data confirmed the existence of the FlaA/MjShoc2/Stat/Ctl-Alf axis.

### Erk is crucial for FlaA/MjShoc2/Stat signaling

**Fig 7:**
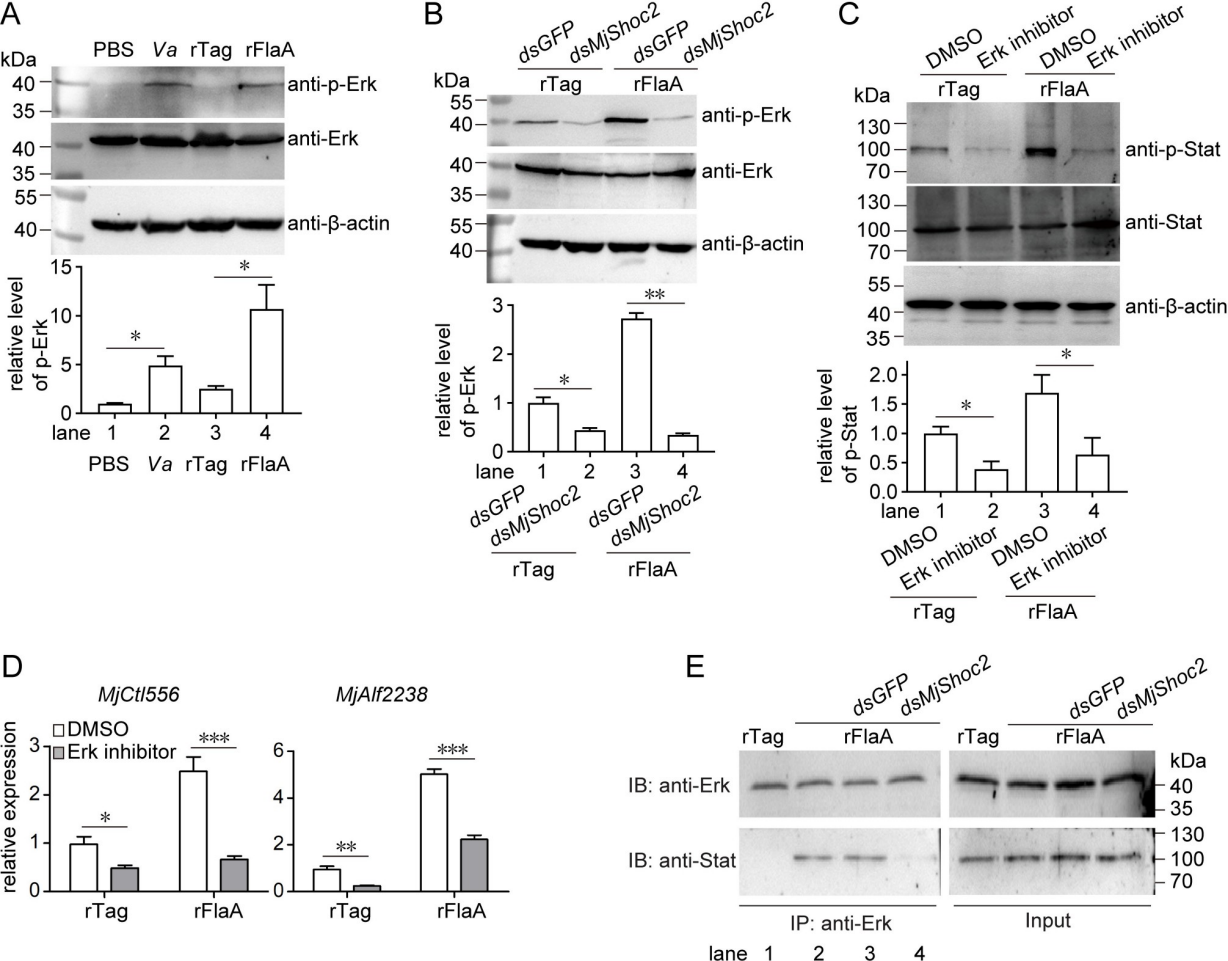
The involvement of Erk in FlaA/MjShco2/Stat signal transduction. (A) Erk phosphorylation induced by *V*. *anguillarum* and rFlaA, with PBS or rTag as control, respectively. The samples were collected at 6 h after injection. Upper panel, representative western blotting result; lower panel, quantification and statistical analysis of three independent repeats. (B) Influence of *MjShoc2* knockdown on Erk-phosphorylation caused by FlaA. rFlaA or rTag injection was performed 24 h after dsRNA application, and Erk phosphorylation was detected 6 h later. Upper panel, representative western blotting result; lower panel, quantification and statistical analysis of three independent repeats. (C) Influence of Erk inhibition on Stat-activation caused by FlaA. The Erk inhibitor was injected into shrimp, and rFlaA was injected at 1 h later. Stat phosphorylation was determined after another 6 h. Upper panel, representative western blotting result; lower panel, quantification and statistical analysis of three independent repeats. (D) The influence of Erk inhibition on the induction of *MjCtl556* and *MjAlf2238* by FlaA. rFlaA injection was performed 1 h after Erk inhibitor application, and the expression levels of *MjCtl556* and *MjAlf2238* were detected 6 h later. Data are mean ± SD from three repeats. (E) Interaction between Erk and Stat. Erk antibodies were used to do the immunoprecipitation assay, and the resultant samples were detected using Western blotting with indicated antibodies. Data are representative of two independent repeats. *, 0.01 < *p* < 0.05, **, 0.001< *p* < 0.01, ***, *p* < 0.001.

Human SHOC2 is a scaffold protein that accelerates ERK1/2 signal transduction, and SHOC2 is highly conserved from parasites to mammals [25]; therefore, we determined whether shrimp Erk participates in FlaA/MjShoc2/Stat signaling. Firstly, the involvement of Erk in the antibacterial response was detected by assessing Erk phosphorylation after *V*. *anguillarum* infection or FlaA challenge. As shown in Fig 7A, both treatments induced Erk phosphorylation. Then, whether this induction was influenced by *MjShoc2* knockdown was analyzed. The data showed that FlaA-induced Erk phosphorylation (Fig 7B, lane 3 *vs* lane 1) was suppressed significantly when *MjShoc2* was silenced (Fig 7B, lane 4 *vs* lane 3), suggesting that MjShoc2 is essential for FlaA-induced Erk phosphorylation.

The contribution of Erk on Stat activation was next detected. As shown in Fig 7C, the Stat activation caused by FlaA (lane 3 *vs* lane 1) disappeared when Erk activity was inhibited using a specific inhibitor (lane 4 *vs* lane 3). Moreover, FlaA-induced expression of antibacterial effectors was also suppressed when Erk inhibitor was applied (Fig 7D). These data suggested that Erk functions upstream of Stat. Afterwards, whether there is an interaction between Erk and Stat was investigated. As shown in Fig 7E, Stat could be detected in the immunoprecipitates of Erk only after FlaA challenge, but not the control challenge (lane 2 *vs* lane 1). In addition, when MjShoc2 expression was pre-silenced, the amount of Stat from the immunoprecipitates decreased (lane 4 *vs* lane 3). These data suggested that Erk was essential for FlaA/MjShoc2/Stat signaling, by acting downstream of FlaA/MjShoc2 and upstream of Stat.

**Fig 8:**
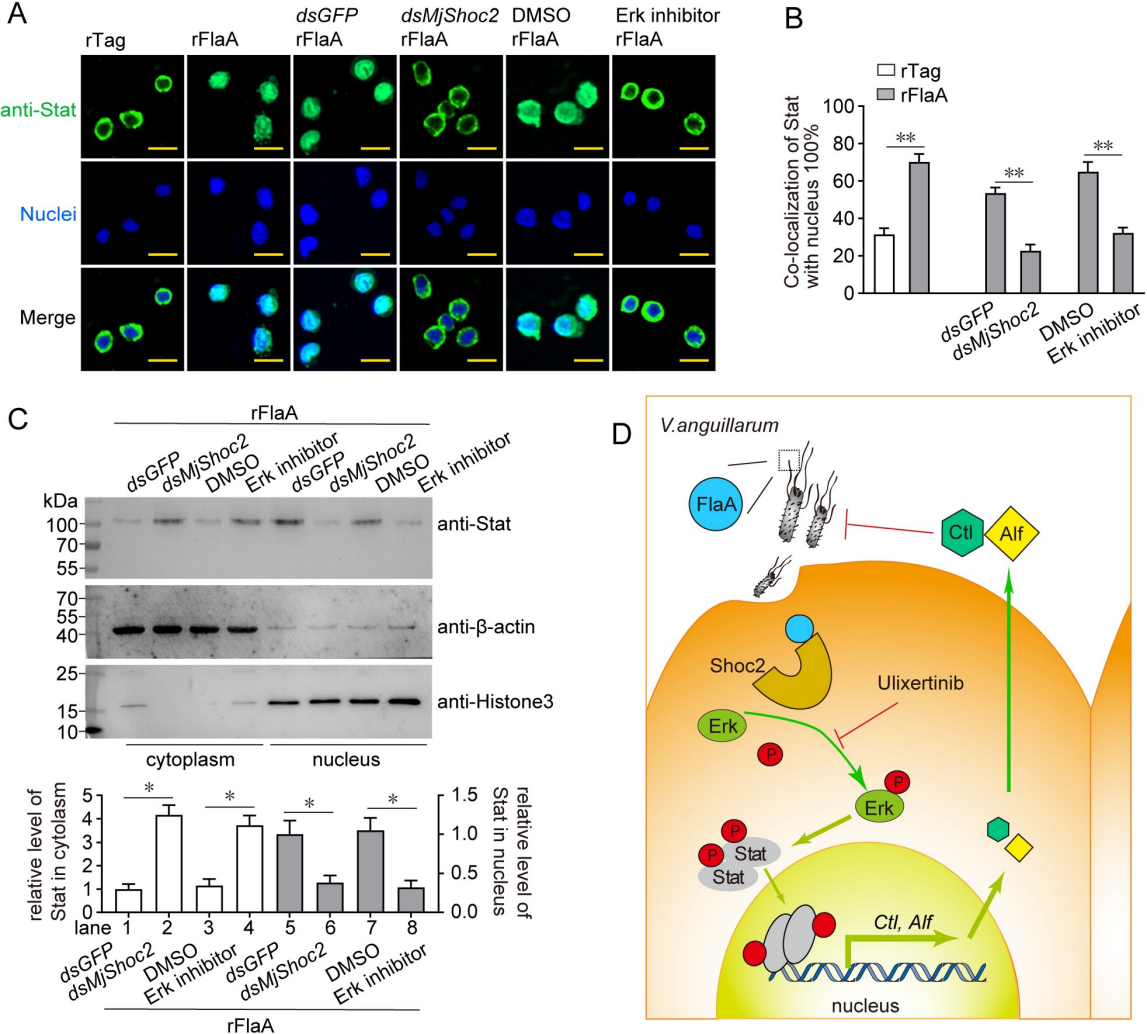
Induction of Stat translocation into the nucleus by FlaA/MjShoc2/Erk axis. (A) Stat translocation analyzed using immunocytochemistry. rFlaA was injected into untreated shrimp, or shrimp pre-treated with dsRNA or Erk inhibitor. Hemocytes were collected at 6 h after rFlaA injection and analyzed with anti-Stat antibodies. DAPI was used to stain the nuclei. Scale bar = 10 μm. Data rare representative of trice repeats. (B) Digitization of the result in (A). The co-localization percentage of Stat and nuclei was analyzed using WCIF ImageJ software. (C) Distribution of Stat after treatment. β-actin and histone 3 were detected as internal references for the cytoplasmic and nuclear proteins, respectively. Upper panel, representative western blotting result; lower panel, quantification and statistical analysis of three independent repeats. Twenty animals were pooled for each sample. *, 0.01 < *p* < 0.05. (D) Model of the FlaA/MjShoc2/Erk/Stat signaling axis. MjShoc2 recognizes the *V*. *anguillarum* FlaA, and induces the phosphorylation of Erk, which leads to Stat activation. Nuclear-located Stat transcriptionally regulates the expression of MjCtl556 and MjAlf2238, which act as the direct antimicrobial effectors in the antibacterial response.

Next, whether Erk determines Stat activation after FlaA challenge was studied by determining Stat’s subcellular location. As shown in Fig 8A and 8B, FlaA challenge induced the enrichment of Stat in the nucleus. However, both *MjShoc2* knockdown and Erk inhibitor application suppressed this enrichment, suggesting that MjShoc2 and Erk were crucial for FlaA-induced Stat activation. This result was confirmed by separating and analyzing the cytoplasmic and nuclear proteins after FlaA challenge in the *MjShoc2* knockdown shrimp or Erk inhibitor-treated shrimp. As shown in Fig 8C, the amounts of Stat were much higher in the experimental samples than in the control samples (lane 2 *vs* lane 1, lane 4 *vs* lane 3) in the cytoplasm, and lower in the experimental samples than in the control samples (lane 6 *vs* lane 5, lane 8 *vs* lane 7) in the nucleus, suggesting that the FlaA-induced translocation of Stat from cytoplasm to nucleus was blocked by *MjShoc2* knockdown or Erk inhibition. Taken together, these data supported the presence of the FlaA/MjShoc2/Erk/Stat signaling axis in shrimp (Fig 8D).

## Discussion

Flagellin is a key virulence factor for both gram-positive and gram-negative bacteria because of its involvement in flagellar motility and other pathogenicity-related properties [26,27]. Flagellin can stimulate the host defense and the expression of antibacterial effectors in multiple organisms, including insects, mammals, and plants [8,28,29]. Moreover, flagellins from different bacteria share conserved N and C-termini [30]. Thus, flagellin is a good ligand for detection by the host innate immunity. Sensing flagellin and mounting a downstream defense response is an important strategy in host antibacterial immunity. For example, mammals recognize the bacterial flagellin through membrane receptor TLR5 and initiate NF-κB signaling-mediated expression of proinflammatory cytokines, including TNF-α and IL-12 [12]. Mice lacking TLR5 are susceptible to the gram-negative bacterium *Salmonella typhimurium*, a common pathogen causing the acute food-borne gastroenteritis, and *Pseudomonas aeruginosa*, the most common pathogen causing hospitalized respiratory infection [31]. In addition, the plant FLS2 from *Arabidopsis thaliana* recognizes flg22, a peptide corresponding to the most conserved domain and the elicitor-active epitope of flagellin [28,32]. *A*. *thaliana fls2* mutants showed a high sensitivity to the pathogen *Pseudomonas syringae*, one of the most ubiquitous plant pathogens, when infected by spraying the bacteria onto leaf surfaces [33]. In the present study, we discovered that MjShoc2, a newly identified flagellin-recognizing protein in shrimp, stimulated the Erk/Stat-mediated expression of immune effectors after recognizing flagellin. Silencing *MjShoc2* expression led to increased mortality and more serious tissue damage after infection by *V*. *anguillarum*, the causative agent of Vibriosis, which is the most serious bacterial disease in shrimp aquaculture. This suggested that discrimination of bacterial pathogens by recognition of flagellin is a general antibacterial strategy among invertebrates, vertebrates, and plants [34].

Previously, we reported another flagellin recognizing protein, Leulectin, in shrimp. In contrast to MjShoc2, Leulectin is a soluble protein and can be detected in the circulating hemolymph. By recognizing flagellin, Leulectin prevents the attachment of *Vibrio* spp. to shrimp cells, thereby inhibiting *Vibrio* colonization [35]. Therefore, Leulectin functions to recognize the extracellular flagellins. Although *Vibrio* spp. are generally regarded as extracellular pathogens, we observed the presence of intracellular FlaA (or FlaA fragments) in shrimp hemocytes after *V*.*anguillarum* infection. We used rFlaA conjugated with a cell-penetrating peptide, which was found able to penetrate through cell membrane and reach the cytoplasm, to imitate the monomeric flagellin present in the cytosol, and found that it could be recognized by MjShoc2 and led to an immune response. Therefore, both Leulectin and MjShoc2 play important roles in antibacterial immunity by recognizing extracellular flagellin and intracellular flagellin, respectively.

The role of MjShoc2 in the antibacterial response was achieved by inducing the expression of certain immune effectors after recognition of flagellin. These effectors include C-type lectins and anti-lipopolysaccharide factors. C-type lectin is characterized by the C-type lectin domain, which mainly binds carbohydrates. This protein family is expressed abundantly in invertebrates, especially in crustaceans. Based on their ability to interact with the carbohydrates exposed on the surface of bacteria, shrimp C-type lectins play important roles in the antibacterial response by promoting hemocytic phagocytosis, regulating microbiota homeostasis, or acting as direct bacteriostatic peptides [24]. Anti-lipopolysaccharides factors are the primary antimicrobial peptides of crustaceans. This family generally bind to LPS and show strong antimicrobial activity mainly against gram-negative bacteria [23]. The induction of these direct immune effectors after recognition of flagellins enable the host to mount a quick response against invading bacteria. Immune effectors’ induction was shown to rely on Stat, which was identified as an important transcription factor in shrimp immunity [36]. Only one Stat is expressed in the crustacean genome, and it shows the highest similarity to STAT5 among the six mammalian STAT homologs. STAT5-responsive elements were identified in the promoter sequences of both *MjCtl556* and *MjAlf2238*, suggesting the possible transcription regulation of these two genes by shrimp Stat. The ChIP assay confirmed the binding of Stat to the *MjCtl556* and *MjAlf2238* promoters, which further demonstrated that flagellin stimulation led to Stat binding. This supported the view that recognition of flagellin by MjShoc2 activates Stat to induce the expression of antibacterial effectors. This view was strengthened by the results that the flagellin which could not be recognized by MjShoc2 in cytoplasm did not induce effectors expression and Stat activation (S4 Fig).

The Stat activation caused by FlaA/MjShoc2 was found to be dependent on Erk phosphorylation. MjShoc2 consists of tandem arrays of LRRs and, like other scaffold proteins with a similar arrangement, adopts a horseshoe-shaped solenoid structure that provides a platform for signaling molecules [25]. By organizing the binding partners into a stable configuration, these scaffold proteins contribute to the specificity and efficiency of signal transduction. A primary function of human SHOC2 is to transduce the signals of the ERK1/2 pathway by tethering Ras and Raf-1 kinase in close proximity after stimulation by growth factors [37]. In addition, SHOC2 recruits enzymes, including the catalytic subunit of the protein phosphatase 1 and E3 ubiquitin ligase HUWE1, into the complex to fine-tune ERK signaling [38,39]. By modulating ERK signaling, SHOC2 is involved in various biological processes, such as embryogenesis, development, and tumorigenesis [40]. The present study showed that MjShoc2 plays a role in the antibacterial response by modulating the Erk signaling after recognition of bacterial flagellins, and thus provides new perspectives on the significance of Shoc2 in immunity. In summary, the present study identified scaffold protein MjShoc2 as a bacterial flagellin (FlaA)-recognizing protein in shrimp. MjShoc2 plays an important role in resisting *V*. *anguillarum* infection by inducing the phosphorylation of Erk, leading to Stat activation and the induction of immune effectors after recognition of FlaA. The identification of the FlaA/MjShoc2/Erk/Stat signaling axis uncovered a new antibacterial mechanism in shrimp and provided new insights into flagellin-induced immunity in invertebrates.

## Materials and methods

### Yeast two-hybrid assay

Yeast two-hybrid assay was performed to identify the FlaA-interacting proteins, and to detect the pairwise protein-protein interaction. The Matchmaker Gold Yeast Two-hybrid System (Clontech, Mountain View, CA, USA; 630495) was used. To identify the FlaA-interacting proteins, a Gateway AD prey library of kuruma shrimp was first constructed by OE Biotech Company (Shanghai, China). Generally, the total RNAs extracted using TRIzol (Invitrogen, Carlsbad, CA, USA; 15596–026) from hemocytes, hepatopancreas, gills and intestine were equally mixed. mRNA was isolated, and cDNA was synthesized using the CloneMiner II cDNA Library Construction Kit (Invitrogen; A11180). The cDNAs were recombined with the pDONR 222 vector using the Gateway BP Clonase II Enzyme Mix (Invitrogen; 1789–020), and the vectors were transformed into *Escherichia coli* DH10B to generate the cDNA library, which was then recombined with pGADT7- DEST vector. The products were transformed into *E*. *coli* DH10B to generate the Y2H library. For screening, pGBKT7 bait vector containing full length cDNA of *V*. *anguillarum FlaA*, together with the pGADT7 prey plasmids from the AD library were transformed into the Y2H Gold yeast cells. The yeast transformants were screened for growth on SD/-Leu/-Trp/-His/X-α-gal (TDO/X) medium. The blue clones were inoculated onto SD/-Leu/-Trp/-His/-Ade/X-α-gal/AbA (QDO/X/A) medium for further screening. The positive clones grown on QDO/X/A medium were recovered, and the prey plasmids were isolated and transformed into bacterial cells for sequencing. To detect the interaction between MjShoc2 and FlaA, full length cDNA of *MjShoc2* was cloned into pGBKT7 bait vector, and full length cDNA of *FlaA* was cloned into pGADT7 prey vector. The bait and prey plasmids were co-transformed into the yeast cells, and the transformants were plated on SD/-Leu/-Trp (DDO) medium. The positive clones on DDO medium were then grown on the selective medium. Protein-protein interaction was recognized in the grown clones on QDO/A medium and blue clones on QDO/X/A medium.

### Recombinant expression and purification

Recombinant His-tagged FlaA was expressed as described previously [35]. To express the FlaA or GST fused with the cell-penetrating peptide Tat (amino acids 49–57) [22], the primers containing both Tat sequence and FlaA sequence were used to construct the recombinant pGEX4T-1 vector (S3 Table). The proteins were expressed as soluble, and purified using affinity chromatography with ProteinIso GST resin (TransGen Biotech, Beijing, China; DP-201) or ProteinIso Nickel-nitrilotriacetic acid (Ni-NTA) Resin. The contamination of endotoxins was removed by adding an additional wash with excess 0.1% Triton X-114 at 4°C before the final elution. This treatment could reduce the endotoxin level in the proteins to lower than 3 EU/mg. Expression and purification of two effector proteins (MjCtl556 and MjAlf2238) were performed similarly.

### Pull-down analysis

Pull-down assay was performed to detect the interaction between FlaA and native MjShoc2. Shrimp hemolymph (20 ml) was extracted into an equal volume of cold anticoagulant (450 mM NaCl, 10 mM KCl, 10 mM EDTA, 100 mM HEPES, pH 7.45). The mixture was centrifuged at 800 × *g* for 8 min at 4°C to obtain the hemocyte pellet. The pellets were washed with phosphate-buffered saline (PBS, 140 mM NaCl, 2.7 mM KCl, 10 mM Na_2_HPO_4_, 1.8 mM KH_2_PO_4_, pH 7.4), and homogenized in PBS (15 ml) thoroughly using a glass homogenizer. The homogenate was centrifuged by 12,000 × *g* for 30 min to obtain the supernatant, which was used as the pool of native MjShoc2. His-tagged FlaA (10 μg) was incubated with shrimp hemocytes homogenate supernatant (15 ml) at 4°C with overnight gentle rotation. Ni-NTA Resin (20 μl) was used to isolate the interacting complex. The resin was washed, and then eluted using 250 mM of imidazole. The final eluate was analyzed using western blotting. The His-tagged Trx expressed by the plain vector was used to control the non-specific binding of MjShoc2 to the tag.

### ITC assay

ITC test was performed with MICROCAL PEAQ-ITC (Malvern). rFlaA (2 mM) was pumped in the syringe, and rMjShoc2 (0.2 mM) was placed into the cell. rFlaA solution (2 μl) was injected into the cell over a period of 150 s at a stirring speed of 750 rpm. The assay was performed at 25°C and lasted for 50 min. Three independent repeats were performed.

### Animals and immune challenge

Healthy kuruma shrimp (*M*. *japonicus*, ~5 g) were obtained from an aquaculture farm in Jimo, Shandong Province, China. All animal-related experiments were approved by the Animal Ethical Committee of Shandong University School of Life Sciences. The animals were maintained in aerated seawater at 22°C and fed commercial diets daily before use. All shrimp were randomly selected for use. *V*. *anguillarum* (American Type Culture Collection (ATCC) 43305) was cultured in Luria-Bertani (LB) medium (3% NaCl, 1% tryptone, 0.5% yeast extract) at 30°C overnight. The bacterial culture was collected and resuspended in sterile PBS. The suspensions were diluted serially and plated onto agar plates to determine the bacterial counts.

To reveal the expression profiles of MjShoc2 after challenge, shrimp were injected with 10 μg of rFlaA into the hemocoel. At specific time points after infection, the hemocyte pellets were collected. The pellets were homogenized using a glass homogenizer in PBS, and the homogenate was centrifuged at 10, 000 × *g* for 10 min at 4°C to collect the supernatant. After determining the protein concentration using a Bradford protein assay kit (Sangon Biotech, Shanghai, China; C503031), the protein samples were then added with sodium dodecyl sulfate polyacrylamide gel electrophoresis (SDS-PAGE) sampling buffer (Sangon Biotech; C508321). The tissues from at least five animals were pooled to prepare a single sample. Three independent experiments were performed.

### Western blotting

Western blotting was used to determine the protein expression levels. Because of the different expression level and antibody titer, separate gels were used for the internal control and tested proteins. Protein samples were separated using 12.5% SDS-PAGE with 100 μg of protein per lane. For the gel to analyze the loading control, 30 μg of protein was loaded per lane. Thereafter, the separated proteins were transferred onto nitrocellulose membranes using semi-dry transfer with a semi-dry blotter (Jim-X, Dalian, China). The membrane was blocked with 3% non-fat milk, and then incubated with the corresponding primary antibodies, followed by secondary antibodies. The signal from the immunoreactive proteins was visualized using the High-sig ECL western blotting substrate (Tanon, Shanghai, China; 180–5001) and the Tanon 5200 Chemiluminescence Imaging System.

Rabbit anti-human SHOC2 polyclonal antibodies (pAbs) (ABclonal, Wuhan, China; A4199) were found to cross-react with shrimp MjShoc2 because of the sequence conservation, and were used to detect MjShoc2 expression. Rabbit anti-human phospho-STAT5A-Y694 monoclonal antibodies (mAbs) (ABclonal; AP0887) and rabbit anti-human phospho-ERK1-T202/Y204 + ERK2-T185/Y187 pAbs (ABclonal; AP0472) were used to detect phosphorylated shrimp Stat and Erk, respectively, because of the conserved phosphorylated sites. Rabbit anti-human Histone H3 pAbs (ProteinTech, Rosemont, IL, USA; 17168-AP-1) were used to detect shrimp H3 expression. Other antibodies recognizing Stat, Erk, β-actin, and FlaA were generated in the laboratory by immunizing rabbits using the recombinant proteins, and were used at 1: 400 dilution. Mouse anti-His antibody (Zhongshan Bio-Tech, Beijing, China; TA-02), horseradish peroxidase (HRP)-conjugated goat anti rabbit secondary antibody (Zhongshan Bio-Tech; ZB-2301) and HRP-conjugated goat anti mouse secondary antibody (Zhongshan Bio-Tech; ZB-2305) were used at 1:10,000 dilution.

### Quantitative real-time reverse transcription PCR

Quantitative real-time reverse transcription PCR (qRT-PCR) was used to determine the gene expression levels using iQ SYBR Green Supermix (Bio-Rad, Hercules, CA, USA; 170–8882). The primers used are listed in S3 Table. The CFX96 Real-Time System (Bio-Rad) was used to carry out the PCR program: 95°C for 10 min; 40 cycles at 95°C for 15 s, 60°C for 50 s, and plate reading at 72°C for 2 s; and then a melting period from 65 to 95°C. The data were processed using the 2^−ΔΔCT^ method.

### RNA interference (RNAi)

RNAi was performed to knockdown the gene expression by application of double stranded RNA (dsRNA) in vivo. Partial DNA fragments were amplified using specific primers that were linked to a T7 promoter, and used as templates to synthesize *dsMjShoc2* and *dsStat* using a T7 RNAi Transcription Kit (Vazyme, Nanjing, China; TR102). The dsRNA specific for green fluorescent protein (*dsGFP*) was synthesized as the control. The dsRNAs were injected into the shrimp hemocoel at a dose of 5 μg per gram of body weight. The RNAi efficiency was determined using western blotting at 1–3 d after dsRNA injection.

### Survival rate analysis

The survival rate was determined to reveal the role of MjShoc2 in resisting bacterial infection. The shrimp were pre-injected with *dsMjShoc2* or *dsGFP*. *V*. *anguillarum* infection (3 × 10^5^ CFU) was performed 24 h later by injecting the bacteria into shrimp hemocoel. The number of surviving animals were monitored for 5 d.

### Histological analysis

Histological analysis was performed to detect the impact of *MjShoc2* knockdown on tissue morphology. Shrimp were infected with 10^5^ CFU of *V*. *anguillarum*. The hepatopancreas were collected and fixed using Davidson’s AFA fixative (30% ethanol, 22% formalin, and 11.5% acetic acid). After fixing for 24 h, the tissue was dehydrated, embedded in paraffin, sectioned at 7-μm thickness and stained with hematoxylin and eosin (H&E). The slides were observed under the BX51 inverted fluorescence microscope (Olympus, Tokyo, Japan) and the images were captured by the DP70 digital camera system (Olympus).

### Bacterial load determination

Shrimp pre-treated with *dsMjShoc2* or *dsGFP* was infected with 2 × 10^5^ CFU of *V*. *anguillarum* which is resistant to several antibiotics including kanamycin, erythromycin and azithromycin. The hepatopancreas were homogenized in PBS 48 h later. The homogenate was plated onto agar plates containing three antibiotics to determine the bacterial load in hepatopancreas.

### Transcriptome analysis

Transcriptome sequencing was performed to identify the genes regulated by MjShoc2 after FlaA challenge. The rTag or rFlaA (5 μg) were injected into the hemocoel of untreated shrimp or the shrimp pre-treated with dsRNA. Each group contained at least 30 animals. The total RNAs from the hemocytes and intestines were sampled at 6 h after protein injection. Three independent repeats were performed. The RNAs were divided into two parts. One part was used for the transcriptome analysis and the other part was preserved for qRT-PCR validation. The commercial sequencing was performed by BGI (Shenzhen, China). Generally, the concentration and integrity of RNAs were determined using the Bioanalyzer 2100 system (Agilent, Santa Clara, CA, USA), and the RNA integrity of each sample was good with the value above 8.0. Equal amounts of RNAs from hemocytes and intestine of each independent repeat were mixed. mRNA was isolated, fragmented and reversed transcribed into first strand cDNA which was then used to synthesize double strand cDNA. The 3′ adenylated cDNA was linked with adaptors. PCR products were denatured to get the single stranded DNA. cDNA library was next sequenced using the BGISEQ-500 system (BGI, Shenzhen, China) with a paired-end sequencing length of 150 bp. The raw reads were filtered to obtain high quality clean reads which were mapped to unigenes. The FPKM (Fragments per kilobase per transcript per million mapped reads) method was used to compare the gene expression in different samples. Only a gene with an FPKM ≥ 2 was regarded as expressed in a sample. Differential expression was accepted with a cut-off of 2-fold change. The Venn diagram analysis was performed using the Dr. Tom analysis platform at BGI, and visualized using an online Venn tool (bioinformatics.psb.ugent.be/webtools/Venn).

### Agglutination and antimicrobial activity test

For agglutination assay, overnight cultured *V*. *anguillarum* were labeled by incubation with 1 mg/ml of fluorescein isothiocyanate (FITC) for 2 h at 25°C, and then collected and resuspended to a final OD600 of 0.5. An equal volume of bacterial suspension and rGST-MjCtl556 solution (15 μg/mL) were mixed and incubated at 28°C for 1 h. Agglutination was observed under BX51 microscope and the images were captured by DP70 digital camera system.

For antimicrobial test, rMjAlf2238 was added into bacterial suspension (10^7^ CFU/ml in PBS) to a final concentration of 25 μM. After incubation at 25°C for 2 h, the mixture was centrifuged at 7000 × *g* for 10 min to obtain the bacterial pellet, which was next washed with and resuspended in distilled water. A drop (2.5 μl) of the suspension was smeared onto freshly cleaved mica and air-dried at 25°C. Multimode Nanoscope V (Bruker AXS, Karlsruhe, Germany) in tapping mode was used to take the atomic force microscopy images for bacterial morphology.

### Analysis of the promoter region of the effectors

The 5’ end of MjCtl556 and MjAlf2238 cDNA was cloned by rapid amplification of cDNA ends using the SMARTer RACE 5’ Kit (Clontech; 634868) according to the manufacture’s instructions. The transcription start sites were determined by integrally comparing and analyzing the cDNA sequence and the transcriptome sequencing dataset. The upstream sequences were obtained from the *M*. *japonicus* genome (GenBank GCA_017312705.1 and GCA_002291165.1), verified by PCR and sequencing, and analyzed using the online PROMO 3.0 tool (alggen.lsi.upc.es/cgi-bin/promo_v3) and JASPAR tool (jaspar.genereg.net).

### Chromatin immunoprecipitation assay

Chromatin immunoprecipitation (ChIP) assay was performed to investigate the transcription regulation of *MjCtl556 a*nd *MjAlf2238* by Stat, using a ChIP Assay Kit (Beyotime, Jiangsu, China; P2078) according to the manufacturer’s instructions. Shrimp were injected with rFlaA or rTag (5 μg). The hemocytes were collected at 6 h post injection, and used as the pool for ChIP. The immunoprecipitates were analyzed using RT-PCR with primers specific for the fragments containing the STAT5 binding sites, which are listed in S3 Table.

### Co-immunoprecipitation (co-IP) assay

co-IP was performed to detect interaction between Erk and Stat. Shrimp hemocytes were homogenized in IP buffer containing 1% NP-40, 50 mM Tris-HCl, pH 7.4, 50 mM EDTA, 150 mM NaCl, and a protease inhibitor cocktail (Merck, Darmstadt, Germany; P8340). The homogenate was centrifuged at 12,000 × g for 10 min, and the resultant supernatant was precleared with Protein A MagBeads (GenScript; L00273), and used as the protein pool of IP. Erk antiserum (30 μl) was incubated with 1 ml of the supernatant with gentle agitation overnight at 4°C. The beads were added to isolate the interacting complex for 1 h with agitation at 4°C. After washing with IP buffer, the immunoprecipitates were eluted by boiling the beads in SDS-PAGE sample buffer, and detected by Western blotting.

### Separation of nuclear and cytoplasmic proteins

To determine the distribution of Stat and MjShoc2, nuclear and cytoplasmic proteins were extracted separately using a Nuclear Protein Extraction Kit (Solarbio, Beijing, China; R0050), according to the manufacturer’s instructions. Generally, shrimp hemocytes from twenty shrimp were pooled, washed with PBS and homogenized in a glass homogenizer with 1 ml of cytoplasmic protein extraction reagent containing 1 mM phenylmethanesulfonyl fluoride (PMSF). The homogenate was processed five times by alternating vortex shaking for 20 s and ice bath immersion for 3 min, and centrifuged at 15,000 rpm for 20 min at 4°C. The resultant supernatant was used as the pool of cytoplasmic proteins. The sediment was washed trice with PBS, resuspended with nucleoprotein extraction reagent containing 1 mM PMSF, and processed another five times by alternating vortex shaking for 20 s and ice bath immersion for 3 min. Thereafter, another centrifugation at 15,000 rpm for 20 min at 4°C was performed. The supernatant was collected as the pool of nuclear proteins. The protein concentration was determined using a spectrophotometer as described above.

### Immunocytochemistry assay

Immunocytochemistry assay was performed to detect the presence of FlaA in hemocytes, or the distribution of Stat. For FlaA detection, shrimp were injected with *V*. *anguillarum* (2 × 10^5^ CFU) or rFlaA (5 μg). At 6 h after injection, the hemolymph was collected into fresh anticoagulant containing 4% paraformaldehyde for fixing for 10 min, and centrifuged to isolate the hemocytes. The hemocytes were washed and resuspended in PBS, and smeared onto poly-L-lysine coated glass slides. The glass slides were dried, and washed three times with PBS. Afterwards, 0.2% Triton X-100 in PBS was added to the slides and incubated for 10 min. After washing three times with PBS, the slides were blocked with 3% bovine serum albumin (BSA) in PBS at 37°C for 1 h. Anti-FlaA antibodies (1,1,000 dilution in 3% BSA) were added to the slides, and incubated overnight at 4°C. Goat anti-rabbit Alexa Fluor 594 (ThermoFisher, Agawam, MA; A-11037, 1:500 diluted in 3% BSA) was added and incubated for 2 h in the dark. The 3,3′-dioctadecyloxacarbocyanine perchlorate (DiO) (Beyotime; C1038) was used to stain the cell membrane, and 4′,6-diamidino-2-phenylindole (DAPI) was added to stain the nuclei for 10 min, and Finally, the slides were washed with PBS, and observed and captured with a Zeiss LSM 900 confocal microscope.

For Stat detection, shrimp were injected with *dsMjShoc2* or an Erk inhibitor (Selleck Chemicals, Houston, TX, USA; S7854), with *dsGFP* or dimethyl sulfoxide (DMSO) as the control, respectively. rFlaA or rTag (5 μg) were then injected into the untreated or pre-treated shrimp at 24 h after dsRNA injection or 1 h after inhibitor injection. For staining, anti-Stat antibodies (1:1,000 dilution in 3% BSA) and the goat anti-rabbit Alexa Fluor 488 (Abbkine, Wuhan, China; A23220, 1:1000 diluted in 3% BSA) were used. The slides were observed and captured under the Olympus SpinSR10 confocal microscope.

### Quantification and statistical analysis

ImageJ software (NIH, Bethesda, MD, USA) was used to quantify the density of western blotting bands. The relative expression was expressed as the ratio of tested proteins compared to internal control. The co-localization analysis of the immunocytochemistry figures was performed using Wright Cell Imaging Facility (WCIF) ImageJ software. The data for the survival assay were analyzed using the log-rank (Mantel–Cox) test using GraphPad Prism software (GraphPad Inc., La Jolla, CA, USA). The other data were analyzed using Students’ *t* test, and a significant difference was accepted at *p* < 0.05.

## Supporting information

S1 Fig Distribution of MjShoc2 after immune challenge. Shrimp was injected with rFlaA or *V*. *anguillarum*, with rTag or PBS as control, respectively. Hemocytes were collected to separate the cytoplasmic and nuclear proteins. β-actin and histone 3 were detected as internal references for the cytoplasmic and nuclear proteins, respectively. The blotting data are the representative of three independent repeats. At least five animals were pooled for each sample.
(TIF)

S2 Fig Influence of dsRNA application on shrimp survival. Shrimp was injected with dsRNA with a dose of 5 μg per gram of body weight. Survival rate was recorded for 5 d after dsRNA injection. The data was analyzed using the log-rank (Mantel–Cox) test.
(TIF)

S3 Fig Promoter sequence of MjCtl556 and MjAlf2238. The transcription start sites were determined by integrally comparing and analyzing the cDNA sequence and the transcriptome sequencing dataset. The upstream sequences were obtained from the *M*. *japonicus* genome (GenBank GCA_017312705.1 and GCA_002291165.1), verified by PCR and sequencing, and analyzed using the online PROMO 3.0 tool and JASPAR tool.
(TIF)

S4 Fig Influence of rFlaA without Tat peptide on shrimp immune responses. Shrimp was injected with rFlaA (without Tat peptide) with Tag (without Tat peptide) as control. The expression of *MjCtl556* and *MjAlf2238* (A), Stat phosphorylation and Erk phosphorylation (B) was detected. *V*. *anguillarum* was used as positive control for the immune challenge. Each sample originated from at least five animals. The bar charts are shown as the mean ± SD from three independent repeats. The blotting results are representative of three repeats.
(TIF)

S1 Table Candidates of FlaA-interacting proteins screened by Y2H. The positive clones on QDO/X/A medium were selected and the corresponding plasmids were sequenced. The sequences were analyzed by online Blastx tool (blast.ncbi.nlm.nih.gov/Blast.cgi), and the top hit for each sequence was selected.
(DOCX)

S2 Table List of FlaA/MjShoc2-regulated genes obtained by transcriptome screening. The sequences obtained through Venn analysis (rFlaA vs rTag, upregulated; dsMjShoc2 rFlaA vs dsGFP rFlaA, down-regulated; FPKM ≥ 2, fold change ≥ 2) were analyzed by online Blastx tool, and the top hit for each sequence was selected.
(DOCX)

S3 Table Primers used for this study. The restriction enzyme sites were underlined.
(DOCX)
