# Supplementary figures and images for: Shoc2 recognizes bacterial flagellin and mediates antibacterial Erk/Stat signaling in an invertebrate
Source: PLoS Pathog. 2022 Jan 24;18(1):e1010253. doi: 10.1371/journal.ppat.1010253 (PMC8812994; doi:10.1371/journal.ppat.1010253)

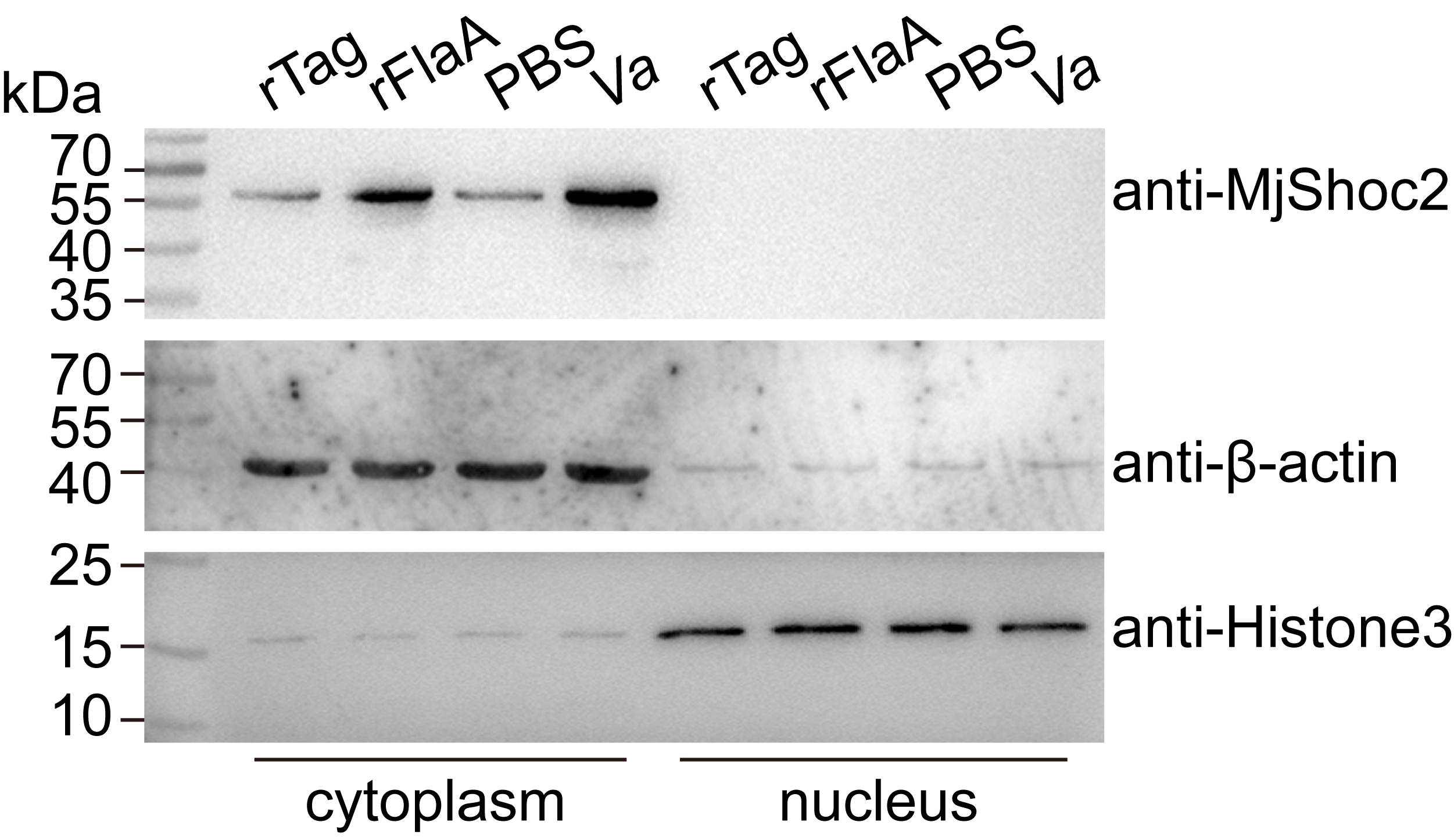

Supplement: S1 Fig — Shrimp was injected with rFlaA or V. anguillarum, with rTag or PBS as control, respectively. Hemocytes were collected to separate the cytoplasmic and nuclear proteins. β-actin and histone 3 were detected as internal references for the cytoplasmic and nuclear proteins, respectively. The blotting data are the representative of three independent repeats. At least five animals were pooled for each sample. (TIF) [file ppat.1010253.s001.tif]

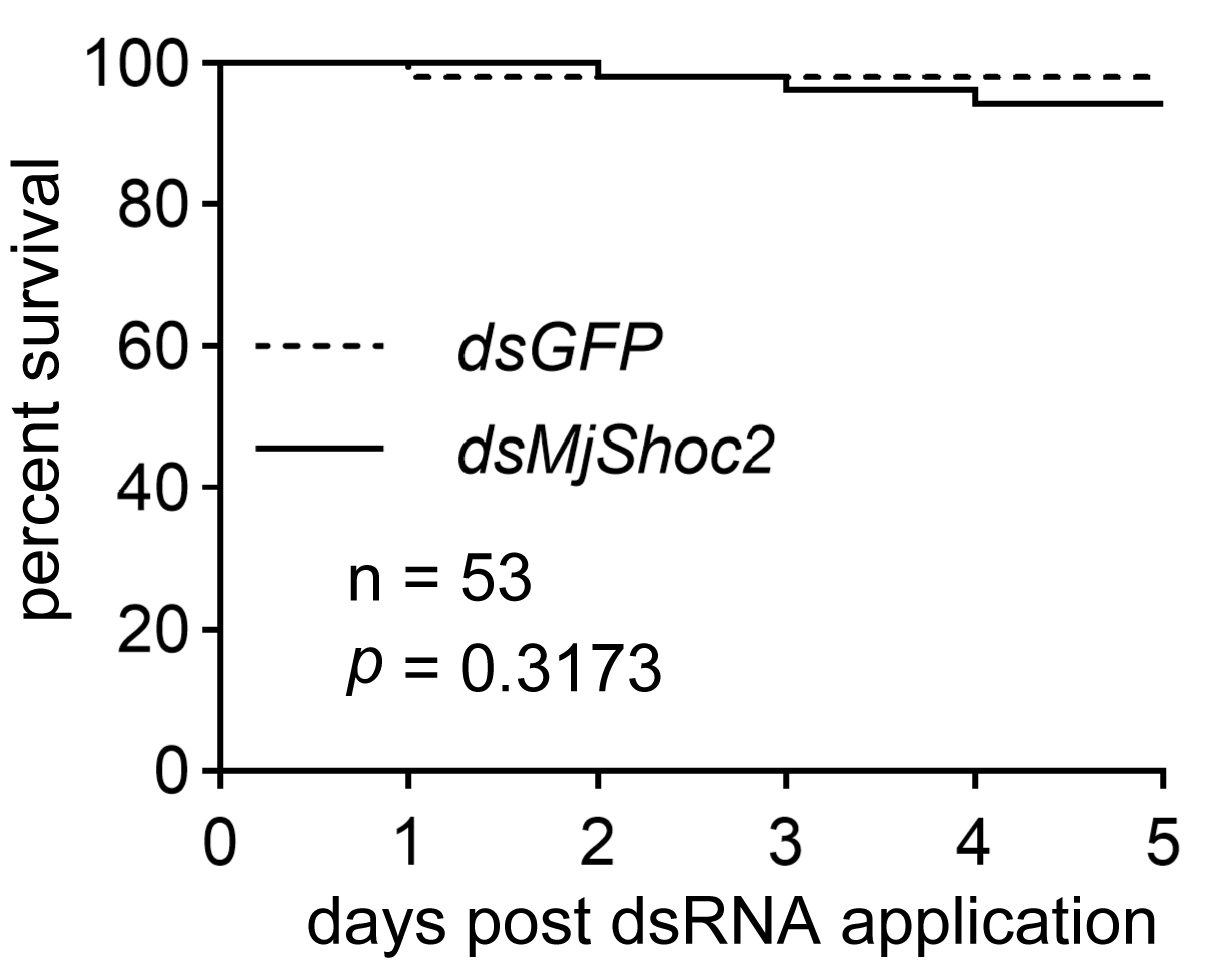

Supplement: S2 Fig — Shrimp was injected with dsRNA with a dose of 5 μg per gram of body weight. Survival rate was recorded for 5 d after dsRNA injection. The data was analyzed using the log-rank (Mantel–Cox) test. (TIF) [file ppat.1010253.s002.tif]

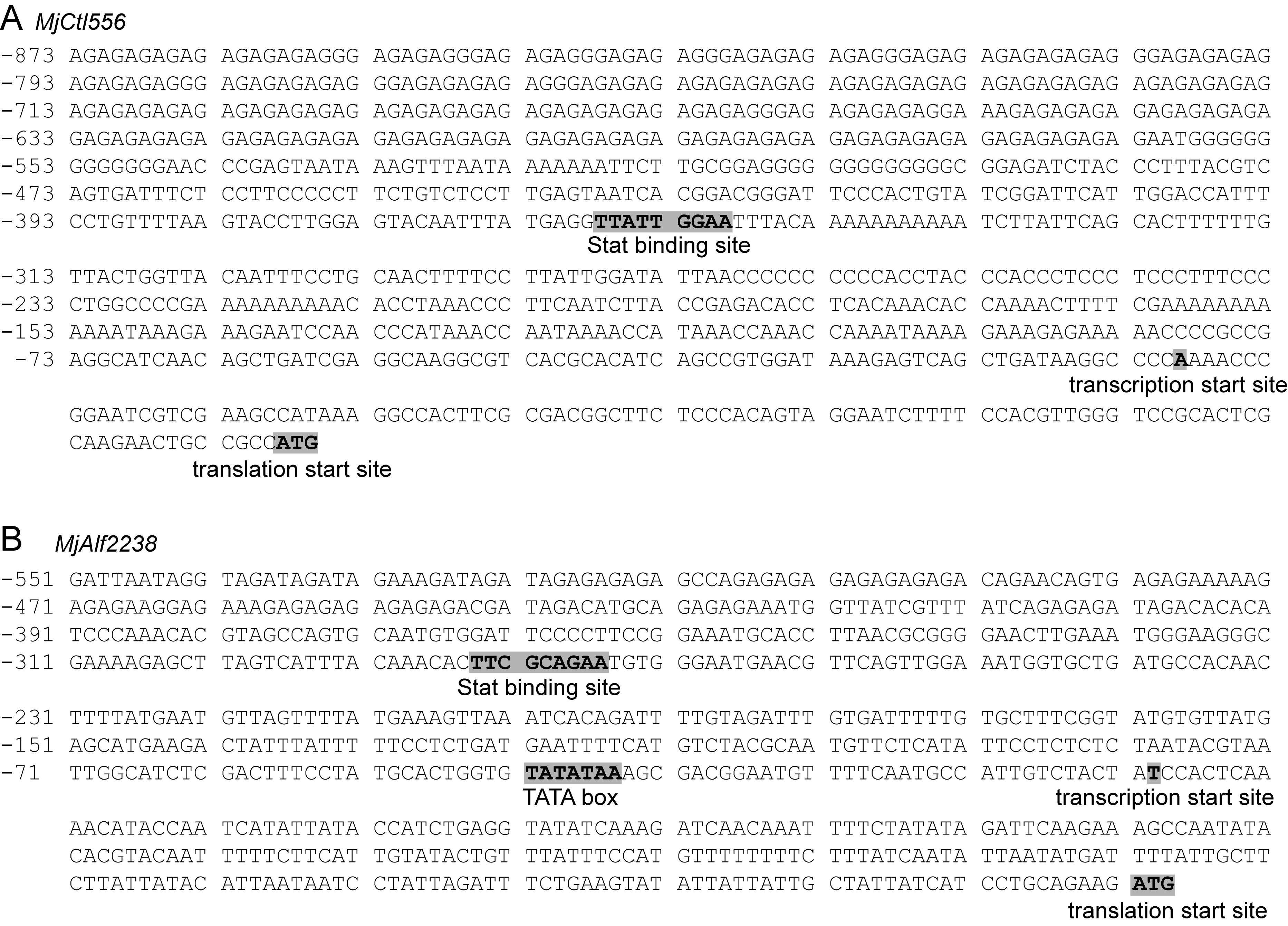

Supplement: S3 Fig — The transcription start sites were determined by integrally comparing and analyzing the cDNA sequence and the transcriptome sequencing dataset. The upstream sequences were obtained from the M. japonicus genome (GenBank GCA_017312705.1 and GCA_002291165.1), verified by PCR and sequencing, and analyzed using the online PROMO 3.0 tool and JASPAR tool. (TIF) [file ppat.1010253.s003.tif]

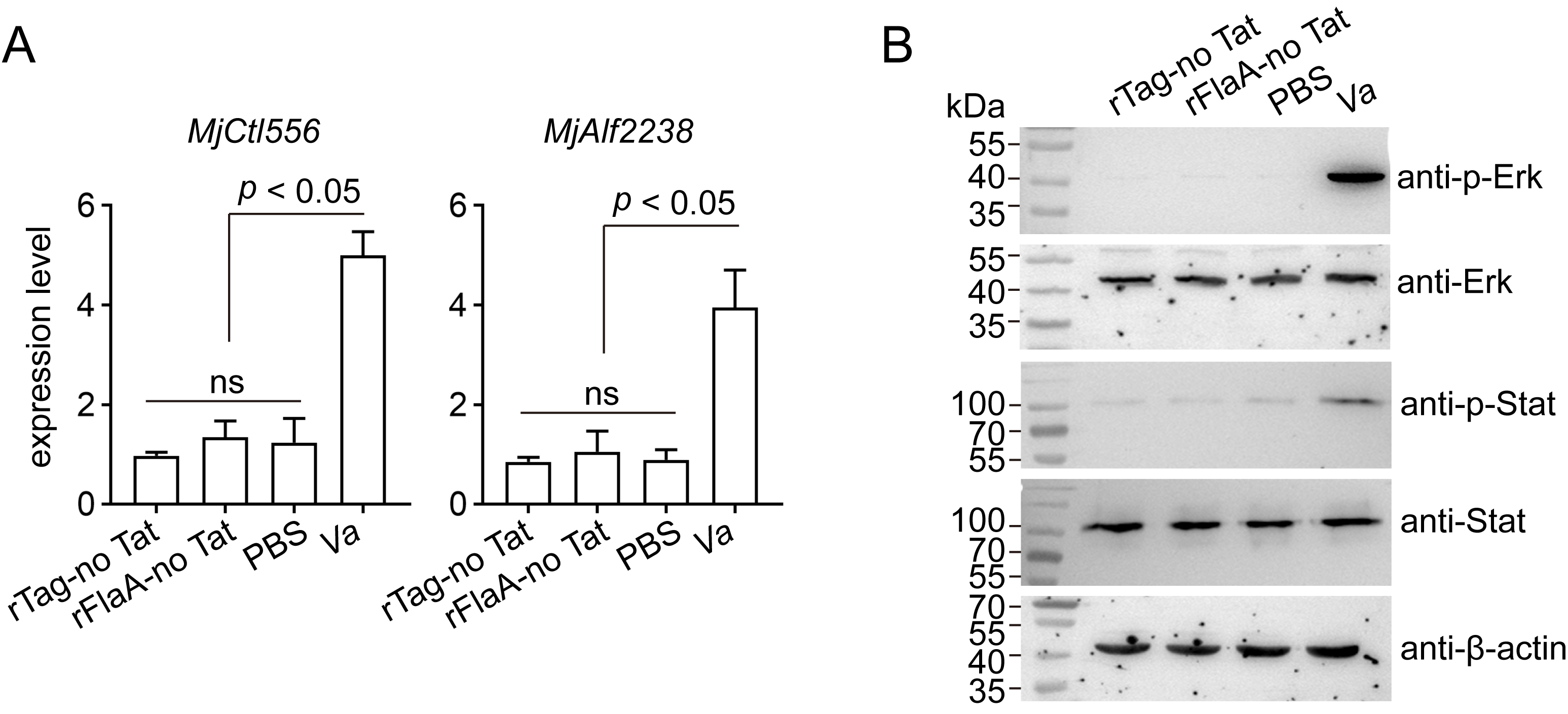

Supplement: S4 Fig — Shrimp was injected with rFlaA (without Tat peptide) with Tag (without Tat peptide) as control. The expression of MjCtl556 and MjAlf2238 (A), Stat phosphorylation and Erk phosphorylation (B) was detected. V. anguillarum was used as positive control for the immune challenge. Each sample originated from at least five animals. The bar charts are shown as the mean ± SD from three independent repeats. The blotting results are representative of three repeats. (TIF) [file ppat.1010253.s004.tif]
