# Supplementary material for: Shoc2 recognizes bacterial flagellin and mediates antibacterial Erk/Stat signaling in an invertebrate
Source: PLoS Pathog. 2022 Jan 24;18(1):e1010253. doi: 10.1371/journal.ppat.1010253 (PMC8812994; doi:10.1371/journal.ppat.1010253)
Supplement: S1 Table — The positive clones on QDO/X/A medium were selected and the corresponding plasmids were sequenced. The sequences were analyzed by online Blastx tool (blast.ncbi.nlm.nih.gov/Blast.cgi), and the top hit for each sequence was selected. (DOCX) [file ppat.1010253.s005.docx]

**S1 Table. Candidates of FlaA-interacting proteins screened by Y2H**

|  | BLAST Hit | GenBank |
| --- | --- | --- |
| a1 | glutamine synthetase [*Penaeus japonicus*] | AWW43688.1 |
| a2 | 40S ribosomal protein S20-like isoform X1 [*Penaeus vannamei*] | XP_027216928.1 |
| a3 | COP9 signalosome complex subunit 5-like [*Penaeus vannamei*] | XP_027237075.1 |
| a4 | leucine-rich repeat-containing protein [*Penaeus japonicus*] | ASR74813.1 |
| a5 | COP9 signalosome complex subunit 5-like [*Penaeus vannamei*] | XP_027237075.1 |
| a6 | 40S ribosomal protein S20-like isoform X1 [*Penaeus vannamei*] | XP_027216928.1 |
| a7 | glucan pattern-recognition lipoprotein [*Penaeus vannamei*] | ROT70455.1 |
| a8 | uncharacterized protein LOC113828749 [*Penaeus vannamei*] | XP_027237560.1 |
| a9 | LOW QUALITY PROTEIN: translocon-associated protein subunit gamma-like [*Penaeus vannamei*] | XP_027232600.1 |
| a11 | cytochrome c oxidase subunit III [*Penaeus japonicus*] | YP_238260.1 |
| a12 | 40S ribosomal protein S20-like isoform X1 [*Penaeus vannamei*] | XP_027216928.1 |
| b1 | uncharacterized protein LOC113803661 [*Penaeus vannamei*] | XP_027210267.1 |
| b3 | COP9 signalosome complex subunit 5-like [*Penaeus vannamei*] | XP_027237075.1 |
| b4 | COP9 signalosome complex subunit 5-like [*Penaeus vannamei*] | XP_027237075.1 |
| b6 | selenium-dependent glutathione peroxidase [*Penaeus monodon*] | AQW41378.1 |
| b7 | uncharacterized protein LOC113808538 [*Penaeus vannamei*] | XP_027215765.1 |
| b8 | histone H3.3 [*Penaeus vannamei*] | XP_027212231.1 |
| b9 | uncharacterized protein LOC113802822 isoform X1 [*Penaeus vannamei*] | XP_027209251.1 |
| b10 | actin, cytoplasmic A3a-like [*Penaeus vannamei*] | XP_027220756.1 |
| b11 | COP9 signalosome complex subunit 5-like [*Penaeus vannamei*] | XP_027237075.1 |
| c1 | uncharacterized protein LOC113803661 [*Penaeus vannamei*] | XP_027210267.1 |
| c2 | 40S ribosomal protein S20-like isoform X1 [*Penaeus vannamei*] | XP_027216928.1 |
| c4 | COP9 signalosome complex subunit 5-like [*Penaeus vannamei*] | XP_027237075.1 |
| c6 | 40S ribosomal protein S20-like isoform X1 [*Penaeus vannamei*] | XP_027216928.1 |
| c7 | 40S ribosomal protein S20-like isoform X1 [*Penaeus vannamei*] | XP_027216928.1 |
| c10 | Marsupenaeus japonicus isolate 14-69 actin mRNA, complete cds | GU645247.1 |
| c11 | COP9 signalosome complex subunit 5-like [*Penaeus vannamei*] | XP_027237075.1 |
| c12 | COP9 signalosome complex subunit 5-like [*Penaeus vannamei*] | XP_027237075.1 |
| d9 | ribosomal protein L10a [*Penaeus vannamei*] | ROT63453.1 |
| d11 | fatty acids binding protein [*Penaeus chinensis*] | ACU82845.1 |
| d12 | putative 60S ribosomal protein L18a-like [*Penaeus vannamei*] | ROT72679.1 |

The positive clones on QDO/X/A medium were selected and the corresponding plasmids were sequenced. The sequences were analyzed by online Blastx tool (blast.ncbi.nlm.nih.gov/Blast.cgi), and the top hit for each sequence was selected.
