# Supplementary material for: Shoc2 recognizes bacterial flagellin and mediates antibacterial Erk/Stat signaling in an invertebrate
Source: PLoS Pathog. 2022 Jan 24;18(1):e1010253. doi: 10.1371/journal.ppat.1010253 (PMC8812994; doi:10.1371/journal.ppat.1010253)
Supplement: S2 Table — The sequences obtained through Venn analysis (rFlaA vs rTag, upregulated; dsMjShoc2 rFlaA vs dsGFP rFlaA, down-regulated; FPKM ≥ 2, fold change ≥ 2) were analyzed by online Blastx tool, and the top hit for each sequence was selected. (DOCX) [file ppat.1010253.s006.docx]

**S2 Table. List of FlaA/MjShoc2-regulated genes obtained by transcriptome screening**

| Function | BLAST hit | GenBank |
| --- | --- | --- |
| Immune response | antilipopolysaccharide factor B1 [*Penaeus japonicus*] | ASR74829.1 |
|  | C-type lectin uncharacterized protein LOC113817858 [*Penaeus vannamei*] | XM_027369962.1 |
|  | Pjchi-3 [*Marsupenaeus japonicus*] | AB008027.1 |
|  | lys-pj for c-type lysozyme [*Marsupenaeus japonicus*] | AB080238.1 |
|  | peritrophin 1 [*Penaeus vannamei*] | QCYY01001877.1 |
|  | Transglutaminase [*Marsupenaeus japonicus*] | AB162767.1 |
| extracellular matrix | uncharacterized protein matrix metalloproteinase-2-like [*Penaeus vannamei*] | XM_027368613.1 |
|  | membrane metallo-endopeptidase-like 1 isoform X6 [*Penaeus vannamei*] | XM_027379402.1 |
|  | membrane metallo-endopeptidase-like 1 isoform X2 [*Penaeus vannamei*] | XM_027379397.1 |
| metabolic process | cytochrome P450 [*Penaeus vannamei*] | GU969106.2 |
|  | HNH/Endo VII nuclease strain HSOK chromosome 13 [*Oryzias latipes*] | CP020633.1 |
|  | uncharacterized protein Alcohol dehydrogenase [*Labrus bergylta*] | XM_020655243.1 |
|  | gamma-interferon-inducible lysosomal thiol reductase [*Penaeus monodon*] | EU837195.1 |
| transmembrane signaling transduction | Integrin [*Penaeus japonicus*] | LC114983.1 |
|  | natriuretic peptide receptor 3 [*Oreochromis niloticus*] | XM_005461822.4 |
|  | ephrin type-A receptor 4-A-like [*Penaeus vannamei*] | XM_027366196.1 |
| transmembrane transport | Na+/K+-ATPase [*Penaeus monodon*] | DQ399796.1 |
|  | excitatory amino acid transporter [*Neodiprion lecontei*] | AC120348.9 |
|  | glucose-6-phosphate exchanger SLC37A4-like (LOC113800436) [*Penaeus vannamei*] | XM_027351203.1 |
| chromosome component | hitone H1 [*Caenorhabditis remanei*] | XM_003089626.1 |
| cytoskeleton component | Tropomyosin [*Marsupenaeus japonicus*] | AB270630.1 |
| ubiquitination | polyubiquitin 8-like (LOC113807539) [*Penaeus vannamei*] | XM_027358820.1 |
| unknown | uncharacterized protein LOC113803661 [*Penaeus vannamei*] | XM_027354466.1 |
|  | hypothetical protein XENTR_v10013218, partial [*Xenopus tropicalis*] | AAMC04000005.1 |
|  | Unknown | No significant similarity found |
|  | hypothetical protein [*Penaeus japonicus*] | LC349927.1 |
|  | hypothetical protein [*Acinetobacter cumulans*] | WP_216076670.1 |
|  | Unknown | No significant similarity found |
|  | Unknown | No significant similarity found |
|  | Unknown | No significant similarity found |

The sequences obtained through Venn analysis (rFlaA *vs* rTag, upregulated; *dsMjShoc2* rFlaA *vs* *dsGFP* rFlaA, down-regulated; FPKM ≥ 2, fold change ≥ 2) were analyzed by online Blastx tool, and the top hit for each sequence was selected.
