# Supplementary material for: Shoc2 recognizes bacterial flagellin and mediates antibacterial Erk/Stat signaling in an invertebrate
Source: PLoS Pathog. 2022 Jan 24;18(1):e1010253. doi: 10.1371/journal.ppat.1010253 (PMC8812994; doi:10.1371/journal.ppat.1010253)
Supplement: S3 Table — The restriction enzyme sites were underlined. (DOCX) [file ppat.1010253.s007.docx]

**S3 Table. Primers used for this study**

| Primers | Sequence (5’-3’) |
| --- | --- |
| **RT-PCR** |  |
| MjShoc2RTF | TCTTCCCTCACTCTGTCACGC |
| MjShoc2RTR | CCCAAGTTCAGTTCCACCATA |
| MjCtl556RTF | GCCAACAATCGCTTCTACAT |
| MjCtl 556RTR | GCAATCCTTCGGAAATCACT |
| MjAlf2238RTF | AGGTTGTTGGGTTGTGGAGG |
| MjAlf2238RTR | GTGCTGGCTTCCCCTCTG |
| MjStatRTF | GGTCCCAGTTCTGTAAGG |
| MjStatRTR | TAGGCACATTCGGATAAA |
| β-actinRTF | CAGCCTTCCTTCCTGGGTATGG |
| β-actinRTR | GAGGGAGCGAGGGCAGTGATT |
| **RNAi** |  |
| MjShoc2RNAiF | GCGTAATACGACTCACTATAGGGTGCGGGTTATGGTGGATGA |
| MjShoc2RNAiR | GCGTAATACGACTCACTATAGGCCAATGCTTTGTGGTAGGTC |
| MjStatRNAiF | GCGTAATACGACTCACTATAGGGACTTTCCTGCTCCGTTTC |
| MjStatRNAiR | GCGTAATACGACTCACTATAGGGCGTTGGCACTGTTGAGAC |
| GFPRNAiF | GCGTAATACGACTCACTATAGGTGGTCCCAATTCTCGTGGAAC |
| GFPRNAiR | GCGTAATACGACTCACTATAGGCTTGAAGTTGACCTTGATGCC |
| **Recombinant expression** |  |
| MjCt556F | CGCGGATCCGCCCCTGACTCCAGCTGCCC |
| MjCtl556R | CCGCTCGAGCACTGCCACGGGCATAGCCT |
| MjAlf2238F | CGCGGATCCTTCGCCCCGCAGTGCCAGGC |
| MjAlf2238R | CCGCTCGAGTCCGTGAAGCCACTGGTTGG |
| FlaAF | CGCGGATCCATGACCATTACAGTAAATACT |
| FlaAR | CCGCTCGAGTTAGCGCTTCTTACGACGTTGGCGGCGGCGCTGCAATAGTGACATTGCAG |
| GSTF | CGCGGATCCCGCCGCCGCCAACGTCGTAAGAAGCGCTAGATGACCATTACAGTAAATACT |
| **Yeast two hybrid** |  |
| MjShoc2-BD-F | GGAATTCCATATGGAGATGATGAGAAAGACAGCT |
| MjShoc2-BD-R | CGCGGATCCTCACATTGTGCGGTATGGACC |
| FlaA-AD/BD-F | GGAATTCCATATGATGACCATTACAGTAAATACT |
| FlaA-AD/BD-R | CGCGGATCCTTACTGCAATAGTGACATTGC |
| **ChIP** |  |
| MjCtl556chipF | CTTGAGTAATCACGGACGGGA |
| MjCtl556chipR | CCAATAAGGAAAAGTTGCAGGA |
| MjAlf2238chipF | TGGGAAGGGCGAAAAGAG |
| MjAlf2238chipR | CATACCGAAAGCACAAAAATCA |

The restriction enzyme sites were underlined.
